# NPC transplantation rescues sci-driven cAMP/EPAC2 alterations, leading to neuroprotection and microglial modulation

**DOI:** 10.1007/s00018-022-04494-w

**Published:** 2022-07-29

**Authors:** Beatriz Martínez-Rojas, Esther Giraldo, Rubén Grillo-Risco, Marta R. Hidalgo, Eric López-Mocholi, Ana Alastrue-Agudo, Francisco García-García, Victoria Moreno-Manzano

**Affiliations:** 1 Neuronal and Tissue Regeneration Laboratory, Centro de Investigación Príncipe Felipe, 46012 Valencia, Spain; 2 Department of Biotechnology, Universitat Politècnica de València, Valencia, Spain; 3 UPV-CIPF Joint Research Unit Disease Mechanisms and Nanomedicine, Centro de Investigación Príncipe Felipe, 46012 Valencia, Spain; 4 Bioinformatics Unit, Centro de Investigación Príncipe Felipe, 46012 Valencia, Spain

**Keywords:** NPC transplantation, Transcriptomic analysis, Spinal cord injury, cAMP, EPAC2

## Abstract

Neural progenitor cell (NPC) transplantation represents a promising treatment strategy for spinal cord injury (SCI); however, the underlying therapeutic mechanisms remain incompletely understood. We demonstrate that severe spinal contusion in adult rats causes transcriptional dysregulation, which persists from early subacute to chronic stages of SCI and affects nearly 20,000 genes in total tissue extracts. Functional analysis of this dysregulated transcriptome reveals the significant downregulation of cAMP signalling components immediately after SCI, involving genes such as EPAC2 (exchange protein directly activated by cAMP), PKA, BDNF, and CAMKK2. The ectopic transplantation of spinal cord-derived NPCs at acute or subacute stages of SCI induces a significant transcriptional impact in spinal tissue, as evidenced by the normalized expression of a large proportion of SCI-affected genes. The transcriptional modulation pattern driven by NPC transplantation includes the rescued expression of cAMP signalling genes, including EPAC2. We also explore how the sustained in vivo inhibition of EPAC2 downstream signalling via the intrathecal administration of ESI-05 for 1 week impacts therapeutic mechanisms involved in the NPC-mediated treatment of SCI. NPC transplantation in SCI rats in the presence and absence of ESI-05 administration prompts increased rostral cAMP levels; however, NPC and ESI-05 treated animals exhibit a significant reduction in EPAC2 mRNA levels compared to animals receiving only NPCs treatment. Compared with transplanted animals, NPCs + ESI-05 treatment increases the scar area (as shown by GFAP staining), polarizes microglia into an inflammatory phenotype, and increases the magnitude of the gap between NeuN + cells across the lesion. Overall, our results indicate that the NPC-associated therapeutic mechanisms in the context of SCI involve the cAMP pathway, which reduces inflammation and provides a more neuropermissive environment through an EPAC2-dependent mechanism.

**Graphical abstract:**

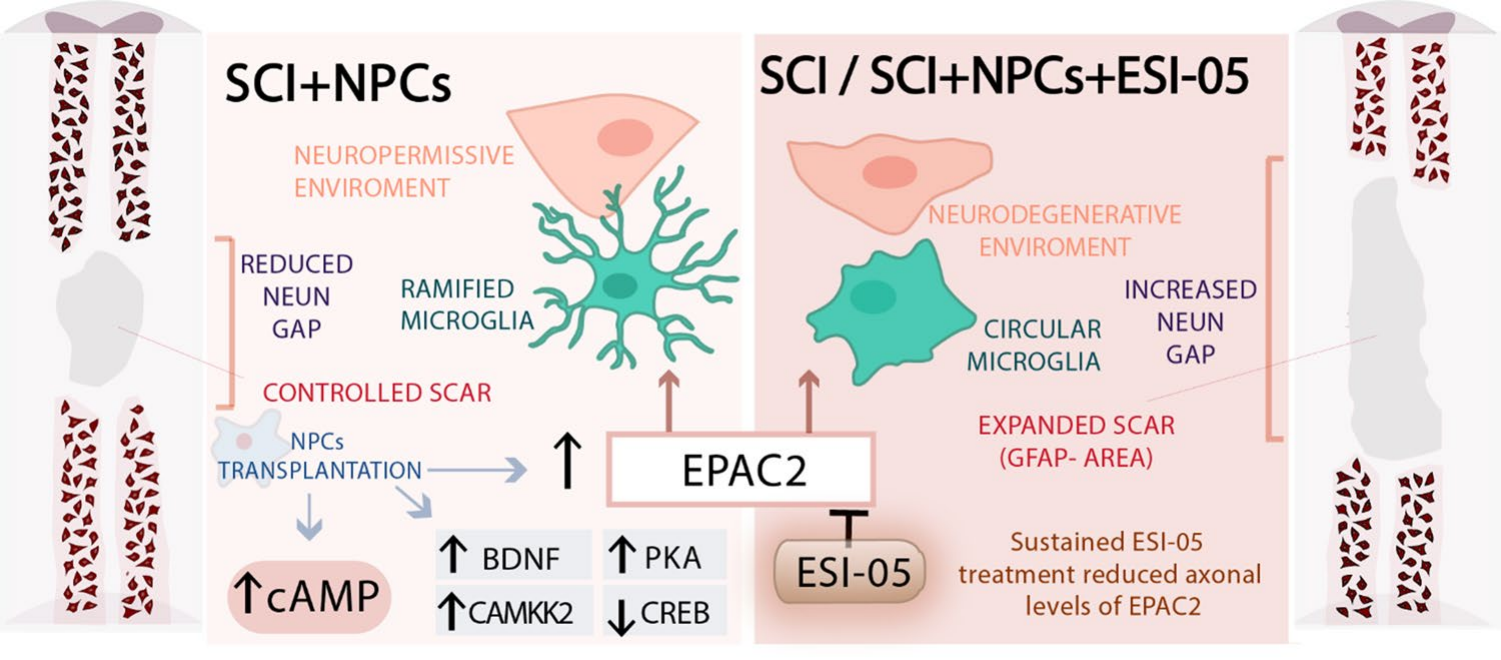

**Supplementary Information:**

The online version contains supplementary material available at 10.1007/s00018-022-04494-w.

## Introduction

Spinal cord injury (SCI) occurs when a traumatic insult disrupts the communication of impulses through the central nervous system (CNS), resulting in the loss of motor and sensory function below the injured area. Even given recent advances, we still lack effective strategies that restore spinal cord functionality after injury [1]. The search for SCI treatments remains challenging, given the highly complex physiopathological events induced by the initial injury and the involvement of multiple cell types and thousands of transcriptional events [2]. Our incomplete understanding of the molecular mechanisms underlying the dynamic pathophysiology of SCI represents a significant impediment to the development of efficient therapeutic strategies and makes the selection of rationalized therapies for acute, sub-acute, and chronic disease states a challenging task.

Cell-based therapies [3] are an attractive alternative to conventional neuronal preservation and regeneration approaches for acute or chronic SCI [4]. Neural progenitor cell (NPC) transplantation represents a promising therapeutic approach, as these multipotent cells can re-establish and replace damaged neuronal circuits (reviewed by Lane, Lepore, and Fischer [5]). The transplantation of adult-derived NPCs obtained from naïve [6] or injured [7] spinal cord tissue has been extensively explored in SCI models; however, the limited availability of NPCs in human adult tissues has hampered clinical translation and prompted the widespread application of NPCs prepared from earlier developmental stages (i.e., from neonatal or foetal tissues) [8].

NPCs differentiate into three different lineages (including neurons and glia) after transplantation into the injured spinal cord. Differentiated neurons can synaptically integrate into the disrupted circuitry and create novel neuronal relays across the injury [9], while glia [10] (astrocytes and oligodendrocytes) provide support to both host and NPC-derived neurons, attenuate glial scar formation [11], and enhance the remyelination of injured axons [12]. NPC transplantation simultaneously provides multiple therapeutic activities and, as such, represents an intrinsically multifactorial therapy. NPCs can (i) serve as a source of new cells to restore the structural integrity of the injured spinal cord [13], (ii) provide neurotrophic support through the secretion of specific factors (such as nerve growth factor, brain-derived neurotrophic factor [BDNF], and glial cell-derived neurotrophic factor) into the injured microenvironment [14, 15], (iii) modulate inflammatory responses [16], and (iv) protect against glutamate-mediated excitotoxicity [17, 18]. Of note, the precise molecular mechanisms driving the NPC-induced pleiotropic spectrum of effects remain minimally defined.

Elevating cyclic adenosine monophosphate (cAMP) levels also represents a promising means of promoting neural regeneration after SCI, as studies have revealed that high levels of cAMP promote axonal growth in a range of species [19, 20]. Throughout development, endogenous levels of cAMP decrease in dorsal root ganglia neurons in parallel with the loss of axon-growing capacity; thus, the decay in basal cAMP levels associates with a switch from the regenerative state of embryonic neurons to the non-regenerative state of the adult CNS [21].

Unfortunately, cAMP levels decay by around four-fold after SCI, thus generating a non-conducive environment for CNS regeneration [22]. Pearse et al. reported that cAMP levels decayed by 64.3% in the spinal cord rostral to the injury, 68.1% in the brainstem, and 69.7% in the sensorimotor cortex for at least 2 weeks after SCI [20]; however, the mechanism driving cAMP decay and whether decay occurs in all spinal cord cell types or a specific subpopulation remains unclear. Overall, findings from a range of related studies have provided insight into the importance of cAMP signalling during CNS regeneration; therefore, a more profound study of this pathway may provide therapeutic targets for CNS pathologies in general and, more specifically, SCI.

Reports have suggested that the cAMP signalling cascade signals solely through protein kinase A (PKA) activation; however, recent research has demonstrated that cAMP binding activates a guanine nucleotide exchange factor—Rap guanine nucleotide exchange factor (GEF) 4 (RAPGEF4), also known as exchange protein directly activated by cAMP 2 (EPAC2) [23]. Guijarro-Belmar et al*.* demonstrated that an EPAC2 activator (S-220) delivered to ex vivo spinal cord slices modulated the lesion environment, prompted axonal outgrowth by reducing astrocyte/microglial activation, and resulted in significantly better locomotor performance for up to 4 weeks after treatment when applied in vivo [24].

To date, cAMP-targeted SCI treatments have focused on the application of general modulators of the pathway, such as adenylate cyclase (AC) stimulators, phosphodiesterase (PDE) inhibitors [25, 26], or cAMP analogues [27] alone or in combination with cell therapies [20, 28–30]. While many of these studies reported functional recovery, applying more specific drugs may provide a deeper understanding of the individual implications of EPAC2/PKA in driving regeneration in the context of SCI and NPC transplantation.

The present study aimed to define alterations to transcriptional profiles during SCI evolution and the molecular processes underlying NPC-based therapies, focusing primarily on cAMP signalling. Furthermore, we investigated the potential involvement of EPAC2 in NPC transplantation-mediated regeneration after SCI through the in vivo administration of the specific EPAC2 inhibitor ESI-05.

## Results

### SCI induces robust and persistent transcriptional dysregulation

**Fig. 1:**
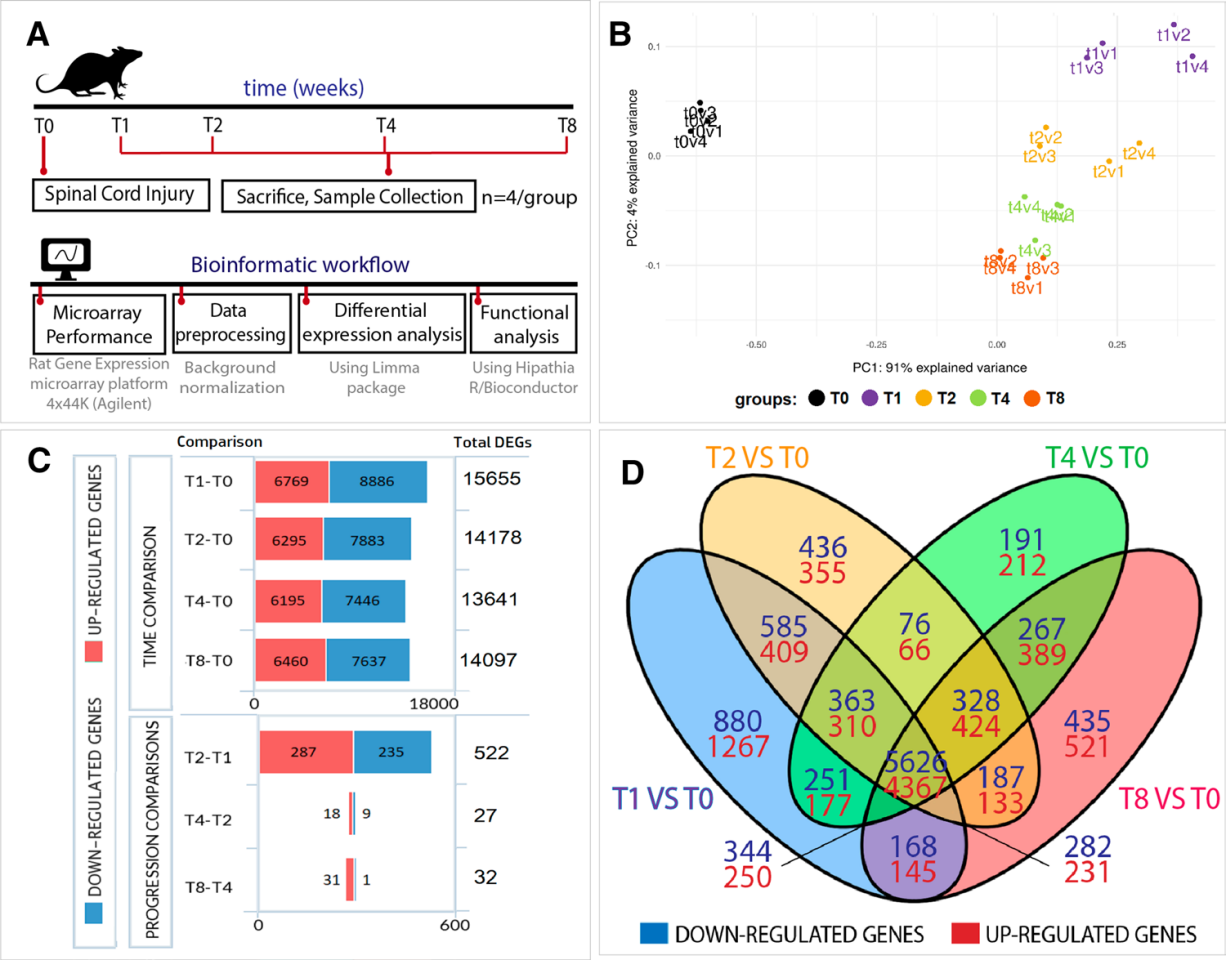
Differential expression analysis during SCI evolution. **A** Schema describing the experimental design, including the in vivo SCI model and the bioinformatic workflow followed in the transcriptional analysis. **B** Principal component analysis (PCA) of our data set, showing clear segregation between the different experimental groups used to analyse the progression of transcriptional alterations after SCI. Please note that v1, v2, v3, v4 denotes the different biological replicates. **C** Graphical representation of DEGs from the indicated comparisons during SCI evolution (genes considered differentially expressed with corrected *p*-values of < 0.05). **D** Venn diagram depicts DEGs for each time point versus the uninjured spinal cord (T0) and overlapping DEGs between comparisons

We evaluated transcriptional profiles at early and chronic stages after SCI to understand the evolving mechanisms occurring after severe traumatic SCI, induced by applying a 250 kdyn contusion at the thoracic vertebrae 8 level in adult Sprague Dawley female rats (Fig. 1A, upper panel displays a schematic diagram of the experimental design). We collected 1.5 cm long samples of the spinal cord tissue (including the injury epicentre) at time points representing early subacute (1 week; T1) [31], late subacute (2 weeks; T2) [32], early chronic (4 weeks; T4), and late chronic (8 weeks; T8) [33] stages to generate transcriptional profiles using the Rat Gene Expression microarray platform (Agilent-014879 Whole Rat Genome Microarray 4 × 44 K G4131F, Agilent). Figure 1A (lower panel) depicts the bioinformatics workflow employed for data processing.

Principal component analysis (PCA) of the samples comprising this data set provided evidence of the clear segregation of the uninjured and injured animals and amongst all time points after SCI (Fig. 1B). While we detected small intra-group dispersion between the animals of the same experimental condition, we observed higher dispersion during earlier stages when compared to later stages (Fig. 1B).

Transcriptional analysis at each time point after SCI provided evidence for robust transcriptional dysregulation compared to the uninjured group (T0), which affected 19,675 differentially expressed genes (DEGs) during SCI progression (representing 55.48% of the rat transcriptome—Online Resource Fig. 1). We observed evident transcriptional alterations from the first week after injury (up to 15,655 genes) that persisted over time, as evidenced by the similar numbers of DEGs observed at 2-, 4-, and 8-weeks post-injury compared to uninjured spinal cords (14,178, 13,641, and 14,097 DEGs, respectively) (Fig. 1C, upper panel). All comparisons provided more downregulated genes than upregulated genes (Fig. 1C); Online Resource Table 1 lists all DEGs for each comparison.

We next performed a differential expression analysis of each stage versus subsequent time points to explore the evolution of SCI, which revealed that the most significant transcriptional differences took place at the earliest stages—between the first and second weeks after injury (522 DEGs)—and a lower number of DEGs between the second to fourth (27 DEGs) and fourth to eighth weeks after injury (32 DEGs) (Fig. 1C, lower panel). These findings suggest that an initial burst of transcriptional activity stabilizes 2 weeks after injury. Furthermore, we discovered that most DEGs (9993 genes) appeared for every time point after SCI when compared to the uninjured spinal cord, as represented by the high number of transcripts at the central intersections of the Venn diagram shown in Fig. 1D. Online Resource Table 2 lists the genes comprising each Venn diagram intersection.

### Functional pathway analysis clusters altered biological functions according to their temporal regulation pattern

**Fig. 2:**
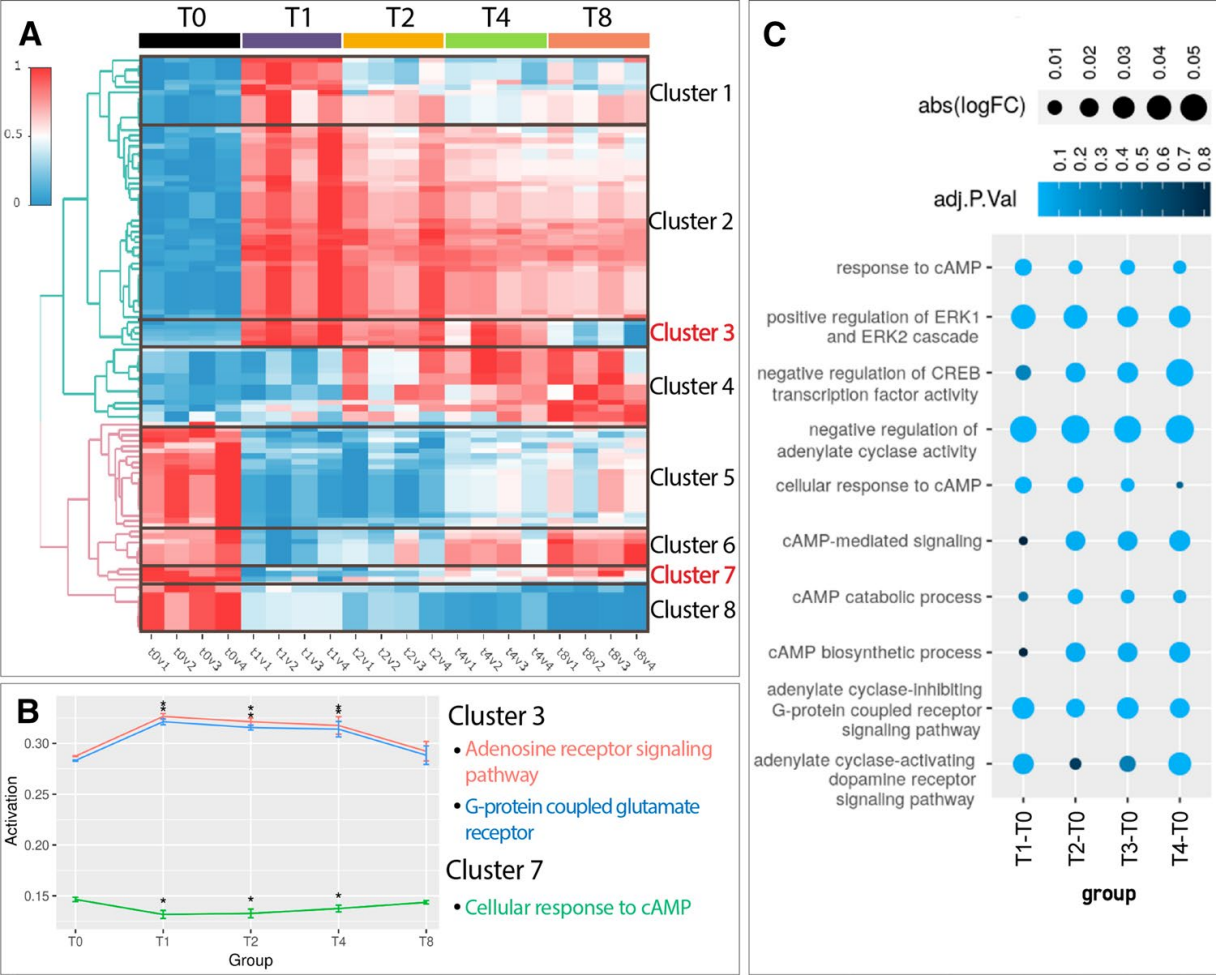
Functional analysis during SCI evolution. **A** Heatmap representing the activation level computed by Hipathia algorithm of differentially activated GO functions for the comparisons on weeks 1, 2, 4, and 8 after SCI relative to the uninjured spinal cord (T0). Red, upregulation; blue, downregulation; v1–v4 indicates replicates for each condition. **B** Graphical representation of the mean activation levels and associated standard deviation for selected functions encountered in clusters 3 and 7 (*, adjusted *p*-value < 0.05). **C** Dot plot depicting significantly altered cAMP-related GO terms after SCI. Colour intensity represents the corresponding adjusted p-value, while dot size indicates the logFC

We functionally analysed the temporal evolution of SCI-induced transcriptomic alterations using the Hipathia algorithm for pathway activity analysis and computed the activation level of each altered function [34]. The functional analysis represented in the heatmap (Fig. 2A, Online Resource Table 3, and interactive heat map in Online Resource File 1) demonstrated the aggregation of altered biological functions into eight different clusters according to their temporal regulation pattern. Functions included in clusters 1–4 become upregulated after SCI, while functions included in clusters 5–8 become downregulated after SCI (Fig. 2B). Figure 2B depicts individual representations of selected functions in clusters 3 and 7 (which display opposite profiles), while Online Resource Fig. 2 depicts selected functions for clusters 1, 2, 4, 5,6, and 8. Online Resource Table 3 describes additional information on cluster composition.

Cluster 3 (Fig. 2A, B) comprises a group of strongly upregulated functions at early stages after SCI (1–4 weeks post-injury) that resolve at later chronic stages (8 weeks after SCI). These functions include the "Adenosine Receptor signalling pathway" (GO:0,060,167) and "G-protein coupled glutamate receptor signalling" (GO:0,007,216). The alteration of these functions may result from the deleteriously high concentrations of ATP [35] and glutamate [36] associated with SCI, which ultimately derive from cell disruption. The upregulation of these functions suggests that these molecules can trigger secondary signalling pathways within the post-injury tissue to induce excitotoxicity by binding to their respective receptors [37].

In contrast to Cluster 3, Cluster 7 (Fig. 2A, B) comprises functions that become downregulated at early stages (from 1 to 4 weeks) after injury but return to pre-injury levels during chronic stages (8 weeks) and includes terms such as "Cellular response to cAMP" (GO:0,071,320). The close temporal coincidence with the upregulated functions in Cluster 3 may indicate that these functions induce the downregulation of Cluster 7 functions. Interestingly, we detected a recurrent representation of cAMP signalling during the exploration of altered functions. We also encountered cAMP-related GO terms among the differentially activated functions (listed in Fig. 2C—indicating the corresponding adjusted p-value and logFC at each temporal comparison). The common overrepresentation of cAMP functions discovered during transcriptional comparisons suggests that SCI profoundly impacts cAMP-related processes.

Cluster 1 comprises a set of functions that became highly upregulated 1 week after injury and then exhibited a slow but progressive downregulation while remaining significantly elevated compared to the uninjured spinal cord. Among these sets of functions, we encountered "Actin cytoskeleton reorganization" (GO:0,031,532) and "positive regulation of JAK/STAT cascade" (GO:0,046,427), with the latter triggering the proliferation and differentiation of multipotent and self-renewing adult stem cells [7] (Sox2/Sox3 + ependymal cells) in response to SCI [38], promoting glial scar formation (preventing secondary damage) [39], and enabling successful axonal regeneration [40]. Previous studies reported that SCI regeneration requires the precise temporal control of JAK/STAT activity, with an early and transient upregulation observed in regenerative species but a slightly delayed and sustained upregulation (at least 30 days) in non-regenerative species [41]. Thus, this temporal profile of JAK/STAT functions may relate to a non-regenerative response.

Cluster 2 includes persistently upregulated functions at all time points after SCI and displays an enrichment in functions related to immunological processes, including "Positive regulation of interleukin-8 production" (GO:0,032,637), "Neutrophil chemotaxis" (GO:0,030,593), "Positive regulation of leukocyte migration" (GO:0,002,687), and "NK T cell proliferation" (GO:0,001,866). The presence of these functions suggests that immunological processes induced by primary injury remain unresolved with time, leading to a chronic inflammatory state (as previously described by Schwab et al. [42]).

Cluster 4 comprises functions that display progressive upregulation over time after SCI and includes terms such as "Cholesterol metabolic process" (GO: 0,008,203), which has been previously linked to SCI and other CNS disorders [43]. Similarly, we encountered additional cholesterol-related processes such as "Cholesterol transport" (GO:0,030,301), which displayed an upward tendency over time (as found in Cluster 6), and "Cholesterol biosynthetic process" (GO:0,006,695), which displayed a downward tendency over time (as found in Cluster 8) [44] among the significantly altered biological functions. Importantly, studies have shown that glutamate excitotoxicity mediates cholesterol-related alterations in CNS injury and disease [45].

### SCI severely alters cAMP signalling

**Fig. 3:**
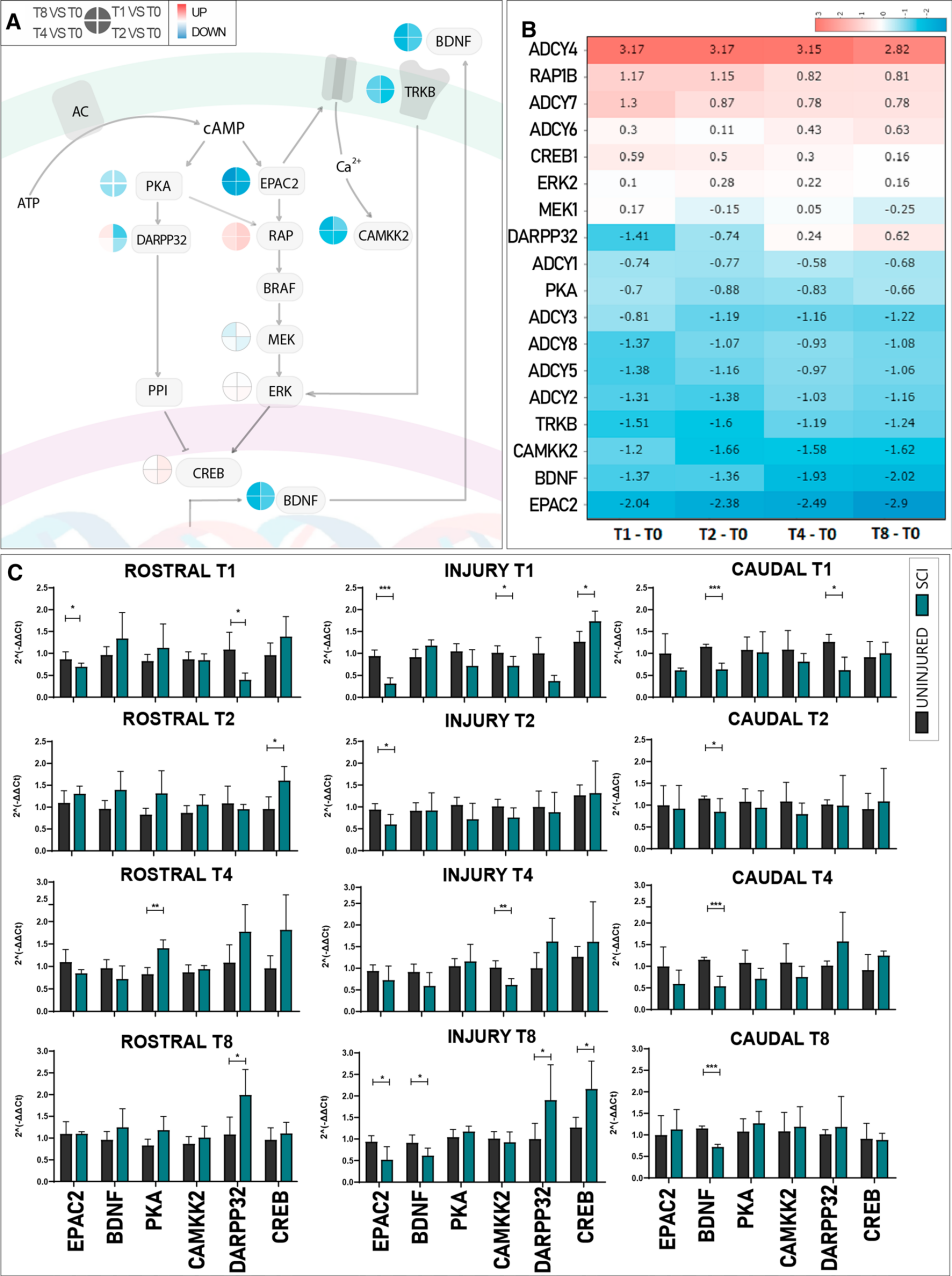
qPCR-mediated Confirmation of transcriptional alterations to the cAMP signalling pathway after SCI. **A** Schematic representation of the cAMP pathway depicting relevant components. The clock icon next to each component represents the evolution of gene expression alterations (from 1 week [T1] to 8 weeks [T8]) as indicated in the legend. **B** Heatmap of cAMP signalling pathway genes showing logFC for each gene over time. Red, upregulation; Blue, downregulation. **C** Microarray expression data validation by qPCR-mediated assessment of gene expression of cAMP-related genes for evolution (from top to bottom) and tissue localization (from left to right) in uninjured spinal cord/control (Black) versus SCI (Green). Data shown as mean ± SEM. Each gene independently assessed for normality using the Shapiro–Wilk test and then analysed using a one-tailed unpaired *t*-student; *p < 0.05, **p < 0.01, ***p < 0.001

We next undertook a deeper evaluation of SCI-induced alterations to cAMP signalling components at the transcriptional level. Figure 3A depicts a schema describing the most relevant components of cAMP signalling and their roles. cAMP signalling pathway initiation occurs after the activation of adenylate cyclases (ADCY), a family of enzymes devoted to cAMP production. cAMP molecules bind and activate the PKA and EPAC effector proteins, which then activate the mitogen-activated protein kinase (MAPK) cascade via Ras-related protein 1 (RAP1) by PKA-mediated protein phosphorylation or the EPAC2-mediated conversion of guanosine diphosphate/guanosine triphosphate [46]. The MAPK cascade culminates with cAMP response element-binding protein (CREB) phosphorylation, which acts as a transcriptional factor to induce the expression of regenerative-associated genes [47]. Such genes include BDNF, an essential axon regeneration modulator that promotes neural plasticity and neurogenesis, exerts pro-nociceptive perception in the uninjured spinal cord, and elicits neuroprotective effects after injury to promote functional recovery [48]. BDNF mediates its effects by binding to tyrosine receptor kinase B (TRKB), which activates the Ras/MAPK cascade through a feedback mechanism [49]. PKA also phosphorylates dopamine- and cAMP-regulated neuronal phosphoprotein (DARPP-32—a complex integrator of dopaminergic signalling) at Thr34 to inhibit the activity of protein phosphatase 1 (PP1) on CREB; however, DARPP32 also acts as a PKA inhibitor when phosphorylated at Thr75 [50]. In myocytes, EPAC2 evokes intracellular Ca^2+^ spikes via calmodulin kinase II (CAMKII) activation and ryanodine receptor (RyR) modulation, thereby linking the cAMP pathway to Ca^2+^ signalling [51].

For all evaluated time points after SCI, we confirmed the significant dysregulation of multiple critical genes involved in the cAMP signalling pathway (as represented in Fig. 3A and shown in an associated heatmap in Fig. 3B [interactive version in Online Resource File 1]) compared to the uninjured spinal cord (T0) when using a false discovery rate (FDR) of < 0.05. We discovered the significant downregulation of ADCY2, 3, 5, and 8 and upregulation of ADCY1, 4, and 6, which agrees with previous observations made in dorsal root ganglia neurons after SCI [52]. We also observed the downregulated expression of the direct cAMP targets PKA and EPAC2 after SCI; however, EPAC2 levels suffered a more robust downregulation than PKA at all evaluated time points, with fold changes ranging from – 2.04 to – 2.89 for EPAC2 and – 0.66 to – 0.88 for PKA in comparison with the uninjured spinal cord (T0). Similarly, we encountered the repression of TRKB2, CAMKK2, and BDNF in all groups after SCI (Fig. 3B, lower section), the upregulation of RAP1B, PPP1CA, and CREB, and the maintained expression of MEK, ERK, and other cAMP-related genes (Fig. 3B). A closer analysis of DARPP32 expression revealed a biphasic behaviour, with downregulated expression occurring at early stages after injury followed by subsequent upregulated expression at chronic stages (Fig. 3B).

We further validated EPAC2, BDNF, PKA, CAMKK2, and CREB transcriptional changes by qPCR analysis of independent samples in rostral (thoracic vertebrae 5–7), injury-affected (thoracic vertebrae 8–9), and caudal (thoracic vertebrae 10–11) segments (1.5 cm long each) (Fig. 3C). The segmental analysis of EPAC2 expression demonstrated significant downregulation at the epicentre of the injury at one (T1), two (T2), and eight (T8) weeks after injury. At week 1, we also encountered significantly downregulated EPAC2 expression within rostral segments (Fig. 3C). BDNF mRNA expression levels significantly diminished caudal to the injury at every time point after SCI compared to the uninjured spinal cord (T0) (Fig. 3C), which agrees with the independent microarray dataset. At chronic stages (T8), BDNF expression became significantly reduced within the injury zone, suggesting an extended lack of neurotrophic factors within more rostral zones. The slight downregulation detected in PKA by the microarray could not be reproduced by qPCR; this discrepancy may arise from the difference in the relative sensitivities of the associated techniques. We found a significant downregulation of CAMKK2 expression at the injury site 1 and 4 weeks after SCI, which partially replicates previous results from the microarray data set. In agreement with microarray data, qPCR analysis replicated the bi-phasic behaviour of DARPP32 expression with an early downregulation at 1 week (rostral and caudally to the lesion site) that became upregulated 8 weeks after SCI (within rostral and injury segments). Meanwhile, we observed upregulated CREB expression 1 week after SCI in the injury site and the rostral region 2 weeks after SCI. Hence, qPCR analysis faithfully replicated most of the transcriptional changes observed in the whole rat spinal cord transcriptional analysis in separate sets of samples. The results provide more evidence for the significant alteration of genes related to cAMP signalling after SCI.

### NPC transplantation rescues SCI-induced alterations to the cAMP signalling pathway

**Fig. 4:**
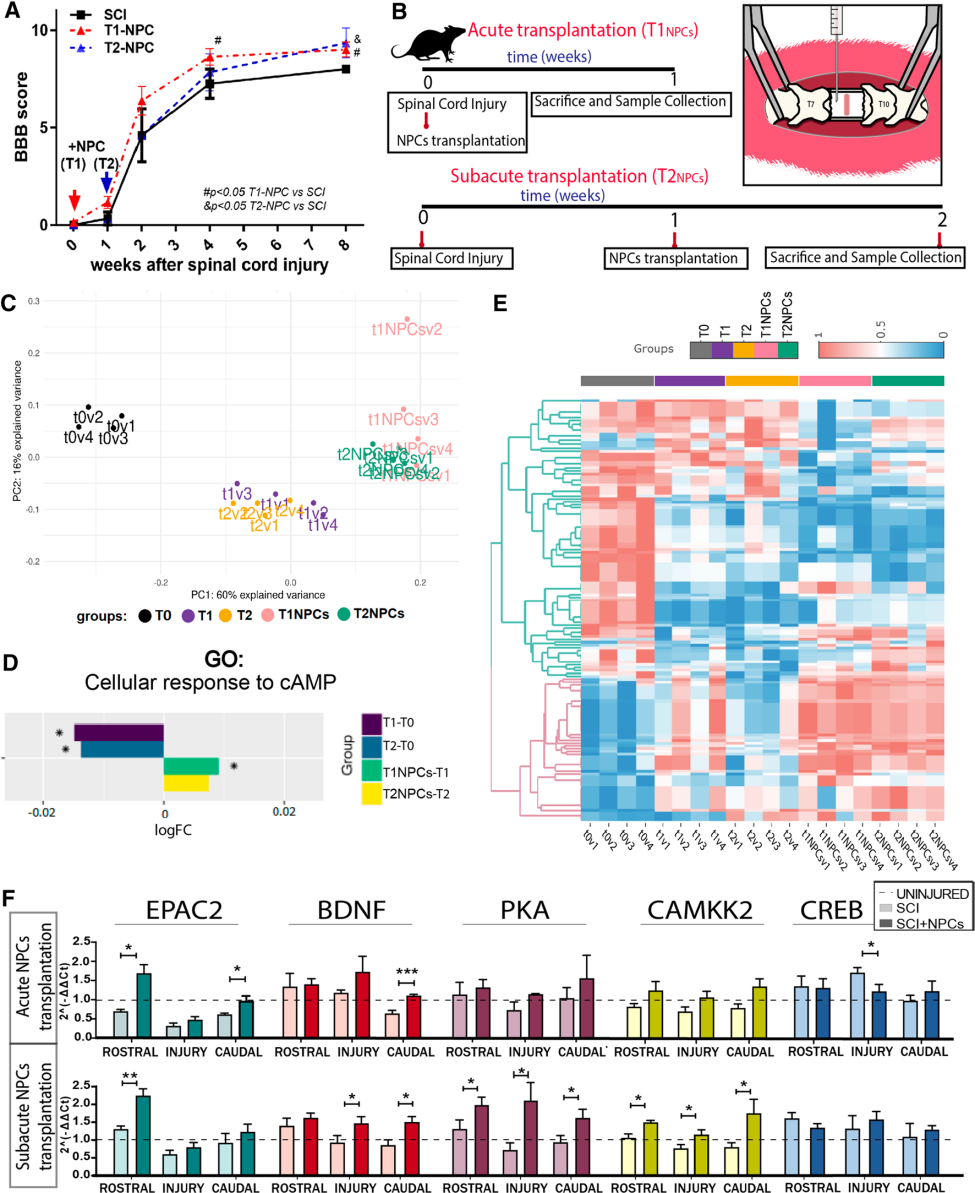
Transcriptional profile analysis after acute/subacute NPC transplantation and analysis of transcriptional changes in cAMP-related genes. **A** Functional locomotor evaluation by open-field BBB scale over 8 weeks post-SCI and after acute or sub-acute NPC transplantation (parallel animals to those used for the microarray analysis). Data expressed as mean ± S.E.M., determined by two-way mixed model ANOVA with Tukey's multiple comparison test (#*p* < 0.05 T1-NPC versus SCI; ^&^*p* < 0.05 T2-NPC versus SCI) (n = 6 animals). **B** Schema describing the in vivo experimental design. **C** Principal component analysis (PCA) of our data set, showing clear segregation between the different experimental groups used to analyse the impact of NPC transplantation on SCI-induced transcriptional alterations; v1–v4 indicates replicates for each condition. **D** Bar-graph representing logFC regarding cellular responses to cAMP for indicated comparisons, showing that NPC transplantation reverses the SCI-induced downregulation in this biological function. **E** Heatmap representing differentially-activated GO functions computed by the Hipathia algorithm, which provided significant results between uninjured (dark grey), injured at week 1 (T1—purple) or week 2 (T2—orange), acute (pink), and subacute (green) NPC transplantation; v1-v4 indicates replicates for each condition (interactive version at Online Resource File 1) **F** qPCR analysis of cAMP-related genes after acute (upper row) and subacute (bottom row) NPC transplantation. Data shown as mean ± SEM, evaluated with the Shapiro–Wilk test to assess normality and one-tailed unpaired t-student or Mann–Whitney test to compare groups: *p < 0,05, **p < 0.01, ***p < 0.001

The functional evaluation of rats receiving NPC transplantation after contusive injury demonstrated significant improvements starting from the second week after therapy (Fig. 4A). Thus, we aimed to transcriptionally evaluate the early impact of NPC therapy on SCI to reveal those mechanisms eliciting later functional recovery. Therefore, we compared the transcriptional profile of animals 1 week after receiving NPC transplants at acute (right after injury) or subacute (1 week after injury) stages after SCI with time-matching non-transplanted animals (Fig. 4B).

PCA analysis showed clear segregation of the different experimental groups, demonstrating consistency between the experimental replicates (Fig. 4C). The differential expression analysis of transplanted versus non-transplanted injured animals revealed that acute NPC transplantation (T1_NPC_) transcriptionally modulated a total of 4227 genes (1933 upregulated and 2294 downregulated), impacting 1032 SCI-related genes (846 early subacute-SCI DEGs and 186 subacute-SCI DEGs) (Online Resource Fig. 3). The subacute transplantation of NPCs (T2_NPC_) modulated a lower number of genes than acute transplantation (3364 genes; 1639 up- and 1725 down-regulated) but had a more significant impact (1281 genes) on SCI-related genes (1019 early subacute-SCI DEGs and 262 late subacute-SCI DEGs). These findings suggest that NPC transplantation at acute and subacute stage SCI significantly impacts transcription in spinal cord tissue; however, subacute NPC transplantation provided a more specific effect regarding the modulation of SCI-induced alterations (Online Resource Fig. 3).

We next functionally analysed the mechanism underlying NPC therapy by applying the Hipathia algorithm to calculate the activation levels of GO biological functions (Fig. 4E). Online Resource Fig. 4 and Online Resource Table 4 depict some functions that reported differential activation levels in injured non-transplanted versus injured NPC transplanted animals. Among the differentially modulated functions, we encountered GO terms related to immunomodulation, such as "Interferon-gamma mediated signalling" (GO:0,060,333), "Positive regulation of interleukin-1 beta secretion" (GO:0,032,731), and "Positive regulation of T-cell proliferation" (GO:0,042,102). We also observed the modulation of neural functions such as "Regulation of short-term neuronal synaptic plasticity" (GO:0,048,172), "Neuron projection morphogenesis" (GO:0,048,812), or "Regulation of cell communication by electrical coupling" (GO:0,010,649) after NPC transplantation. NPC-modulated GO terms also included "Removal of superoxide radicals" (GO:0,019,430), "Ion transmembrane transport" (GO:0,034,220), and "Regulation of generation of L-type calcium current" (GO:1,902,514), suggesting the scavenger function and ionic modulatory capacity of NPCs. Furthermore, NPCs modulated functions related to cell survival ("Negative regulation of mitochondrial membrane permeability involved in apoptotic process," GO:1,902,109) and cell proliferation and differentiation ("Astrocyte differentiation," GO:0,048,708; "Endothelial cell morphogenesis," GO:0,001,886). NPC transplantation also regulated molecular pathways such as "Notch signalling" (GO:0,007,219), "Neurotrophin TRK receptor signalling" (GO:0,048,011), "Cellular response to cAMP" (GO:0,071,320) and "Inositol-phosphate mediated signalling" (GO:0,048,016) (Online Resource Fig. 4).

While focusing on cAMP functions, we discovered that NPC transplantation during acute stages after SCI significantly reversed the downregulation of cAMP cellular responses compared to time-matched injured non-transplanted animals (Fig. 4D). We detected the same trend for subacute transplantation, although this failed to reach significance. We assessed gene expression of cAMP-related targets by qPCR (Fig. 4F) to further characterize the impact of NPC therapy on cAMP signalling. We reproduced the experiment using neonatal NPCs, as a more clinically relevant model, to validate previous high content gene transcriptional results (which employed adult NPC transplantation). We found that acute neonatal NPC transplantation rescued SCI-induced reductions in EPAC2 expression in rostral and caudal segments, while subacute NPC transplantation prompted increased EPAC2 expression compared to the non-transplanted group rostral to the injury. Additionally, NPC transplantation increased BDNF expression in the caudal region when applied at acute or subacute stages compared to injured non-transplanted animals. Furthermore, we found a significant increase in BDNF levels in the injured site for the subacute transplantation group. After sub-acute transplantation, we observed a significant induction in PKA and CAMKK2 mRNA levels throughout the spinal cord in rostral, injury, and caudal regions. While we observed a similar trend for acute transplantation, these results failed to reach significance. Finally, NPC transplantation reduced the upregulated levels of CREB encountered after SCI to control levels. Overall, our results suggest that both adult and neonatal NPC transplantation can correct most SCI-driven alterations in the expression of cAMP signalling components.

### NPC transplantation after SCI increases rostral axonal levels of EPAC2

**Fig. 5:**
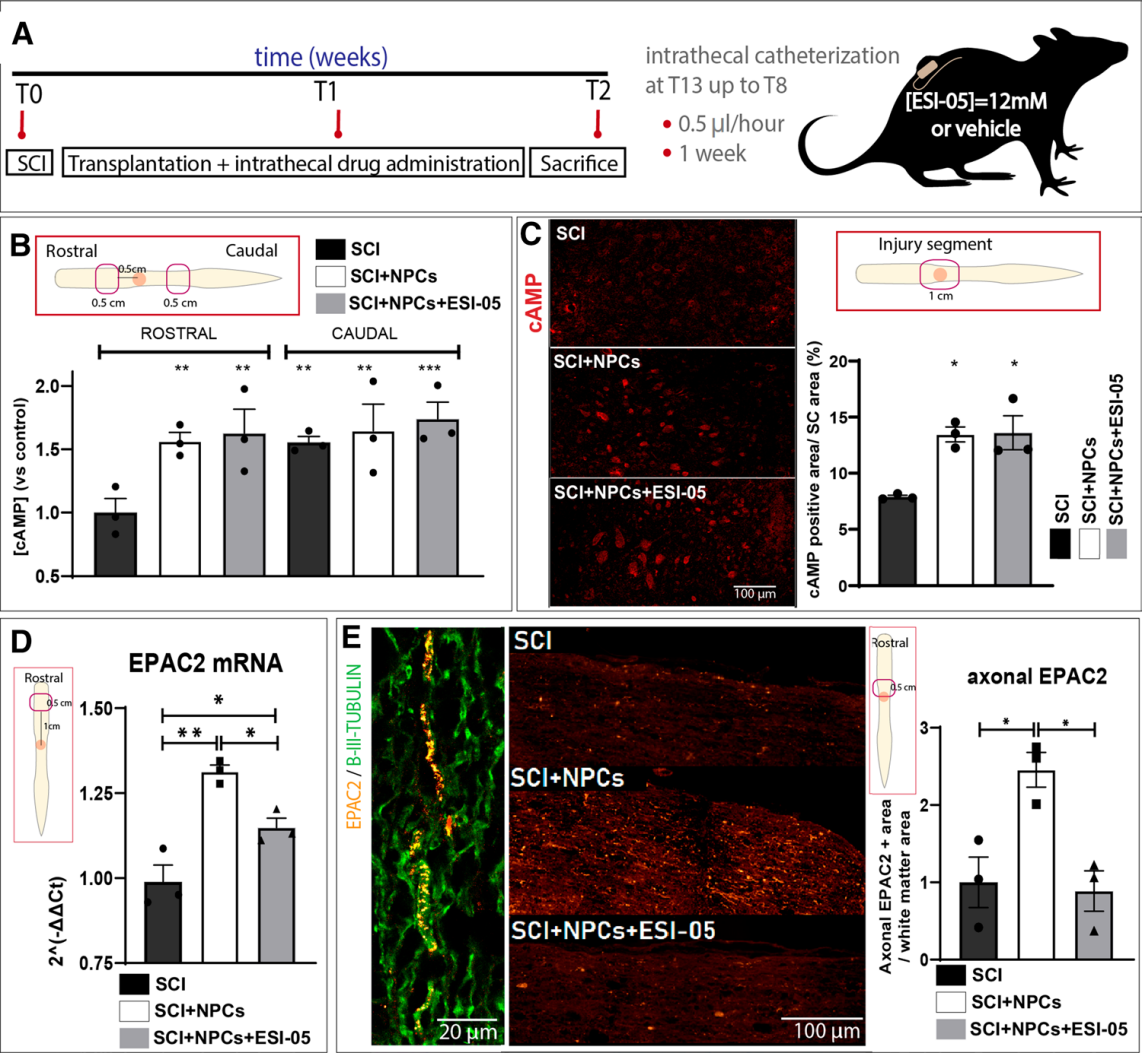
In vivo transplantation of NPCs with the EPAC2 inhibitor ESI-05. **A** Experimental design for the in vivo administration of ESI-05 or vehicle (in SCI and SCI + NPCs groups). Experimental groups: SCI, SCI + NPCs, and SCI + NPCs + ESI-05 (*n* = 3/group). **B** Determination of cAMP concentrations by ELISA immunoassay of the regions rostral and caudal to the injury epicentre relative to injured non-transplanted (SCI) animals. **C** cAMP immunostaining (left panel) and quantification (right panel) in the injured region **D** qPCR analysis of EPAC2 expression in the rostral spinal segment (one-way ANOVA: *p < 0.05; **p < 0.01). **E** Histological visualization of EPAC2 immunostaining (orange) overlapping with B-III-Tubulin + tracts (green) (left panel). Representative images (middle panel) of EPAC2 staining in rostral white matter areas for each experimental condition and its quantification (right panel) (one-way ANOVA: *p < 0.05)

To evaluate the influence of EPAC2 on therapeutic outcomes after NPC transplantation at the sub-acute stage after SCI, we administered a specific EPAC2 inhibitor (ESI-05) alongside NPC transplantation (schema shown in Fig. 5A). The quantification of cAMP concentrations in rostral and caudal spinal cord homogenates (0.5 cm distal from the injury epicentre; Fig. 5B) by ELISA immunoassay revealed that animals with SCI exhibited significantly lower cAMP levels in rostral segments compared to the corresponding caudal segment, which indicates an area-dependent depletion of cAMP induced by SCI (Fig. 5B). Interestingly, NPC transplantation (in the absence and presence of ESI-05) prompted a significant increase in rostral cAMP levels compared to control group, indicating that NPC transplantation inhibits the SCI-induced rostral depletion of cAMP levels. In agreement, anti-cAMP staining in the injured segment revealed a higher percentage of total immunolabeled area in injured animals receiving NPC transplantation (SCI + NPCs) and injured animals receiving NPC transplantation and ESI-05 (SCI + NPCs + ESI-05) compared to untreated injured animals (SCI) (Fig. 5C).

Next, we evaluated the expression of the direct cAMP target EPAC2 in our experimental groups (SCI, SCI + NPCs, and SCI + NPCs + ESI-05). qPCR analysis revealed a significant increase in rostral EPAC2 mRNA levels following NPC transplantation (SCI + NPCs) compared to injured non-transplanted rats (SCI) (Fig. 5D), which agrees with our previous findings (Fig. 4F); however, the addition of ESI-05 (SCI + NPCs + ESI-05) inhibited the NPC-mediated increase in EPAC2 mRNA levels, suggesting that EPAC2 may participate in a positive feedback loop regulating its expression.

Protein immunoreactivity analysis in spinal cord tissues revealed EPAC2 staining within the cytoplasm or plasma membrane of B-III-Tubulin + neuronal bodies in the grey matter and B-III-Tubulin + axonal tracts of the white matter (Online Resource Fig. 5 and Fig. 5E, respectively). We failed to encounter significant differences in the proportion of cytoplasmic or plasma membrane-resident EPAC2 + cells after normalizing data to the total number of EPAC2 + neuronal bodies; however, we observed a significant accumulation of EPAC2 protein in the axonal tracts 0.5 cm rostral to the injury in NPC-transplanted animals (SCI + NPCs) compared to injured non-transplanted animals (SCI) (in agreement with our qPCR data), as supported by a previous study showing elevated levels of EPAC2 only in neurites and growth cones [53]. In contrast, NPC transplantation combined with ESI-05 treatment (SCI + NPCs + ESI-05) reduced the amount of EPAC2 encountered in rostral axonal tracts (Fig. 5E). This result agrees with previous observations showing that EPAC2 inhibition leads to a reduction in the number of vesicles expressing EPAC2 after prolonged synaptic activity, given that EPAC2 activity remains essential for the maintenance of the readily-releasable vesicle pool [54]. Although we failed to detect EPAC2 staining in non-neuronal cells, previous data support the expression of EPAC2 in many other cell types, including astrocytes, ependymal cells, and NPCs [55, 56].

### EPAC2 inhibition reverses NPC-mediated effects resulting in enlarged injury-affected areas

**Fig. 6:**
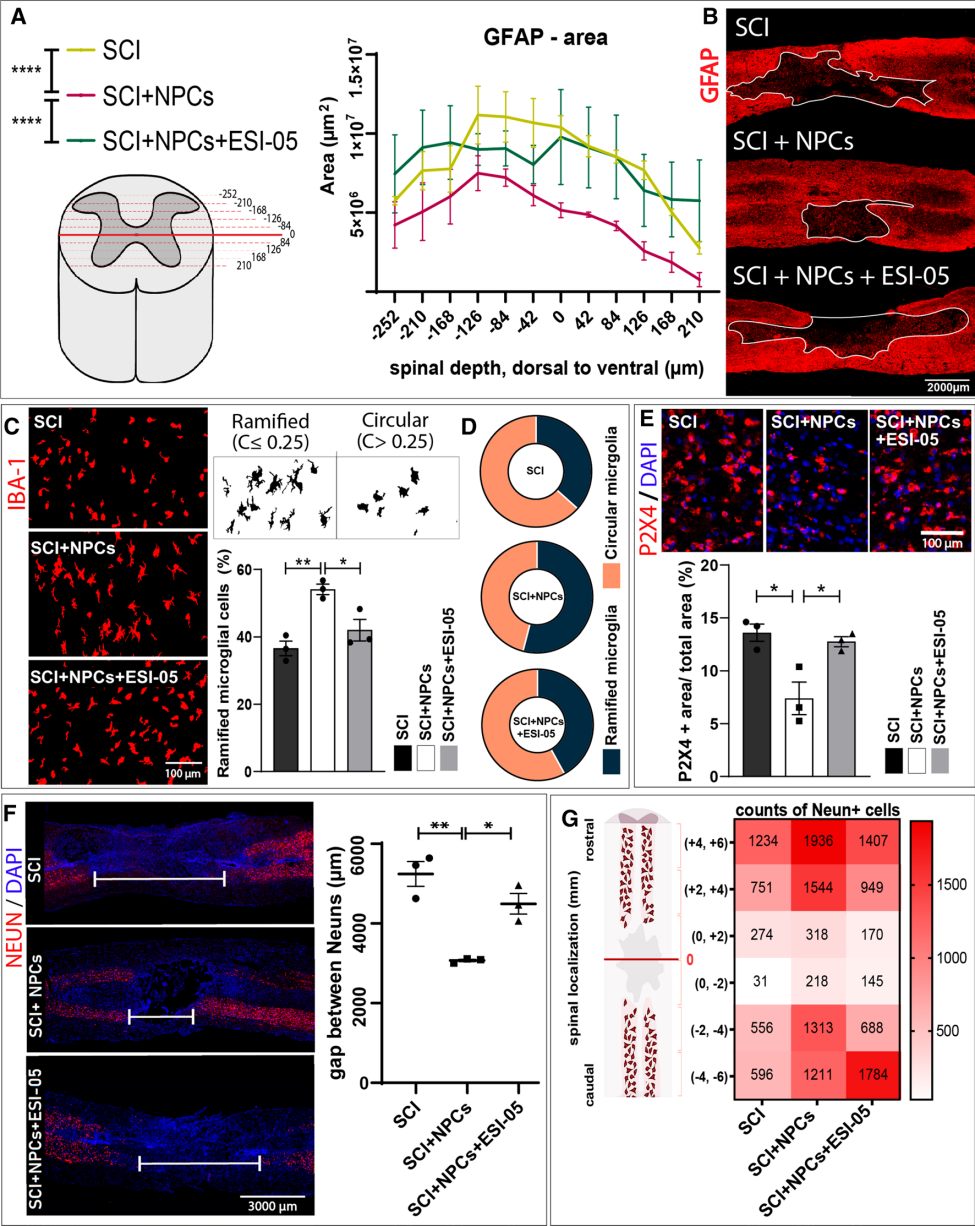
Effects of dual NPC and ESI-05 treatment on scar area, microglial phenotype, and NeuN + cell gap. **A** Quantification of the scar area (GFAP-) throughout longitudinal slices in the mid-region of the injured spinal cord. Two-way ANOVA: ****p < 0.0001. **B** Representative images of GFAP immunostaining and traced GFAP- area (white line). **C** Morphological visualization of microglial cells by Iba-1 staining and determination of the percentage of ramified microglial cells. **D** Pie charts representing the microglial population segregated in ramified or circular cells for each experimental group. **E** Representative images of P2X4 immunostaining and its quantification. One-way ANOVA: *p < 0.05. **F** Representative images (left panel) of the sparsity of NeuN + cells through the injury and quantification of the gap distance (right panel). **G** Heat map showing the mean number of NeuN + cells per experimental group in each spinal segment of 2 mm

We next assessed the influence of NPC transplantation in the presence/absence of ESI-05 during the early stages of scar resolution (2 weeks after injury) by measuring the extent of the area delimited by the astrocytic barrier (the GFAP- area). Analysis of the GFAP- area throughout the central slices of the injured spinal cord revealed that NPC-treated animals (SCI + NPCs) displayed a significantly reduced scar area compared to injured non-transplanted animals (SCI); however, co-treatment with ESI-05 (SCI + NPCs + ESI-05) abolished the effect of NPCs on scar resolution, resulting in the development of a scar area similar to injured non-transplanted animals (Fig. 6A, B). Overall, our results describe a critical role for EPAC2 during the early stages of glial scarring in vivo.

### ESI-05 treatment evokes a phenotypical microglial shift towards amoeboid-like cells reversing the ramified phenotype induced by NPC transplantation

We next evaluated microglial morphology by Iba-1 staining in terms of circularity [57]. Microglia are morphologically dynamic cells that change from a multi-branched "ramified" state under homeostatic conditions to an amoeboid-like shape under pathological conditions [58]. Circular microglia generally display a proinflammatory M1 profile, while ramified microglia have been associated with an anti-inflammatory and neuroprotective M2 state [59]. To focus on the microglial cell population with greater relevance to neuroprotection, we specifically studied the morphology of microglia in white matter regions rich in NeuN + neurons. Furthermore, the study of Iba-1 staining in those specific regions allows the exclusion of macrophages from our analysis since they localize to peri-injury areas. We discovered that NPC transplantation (SCI + NPCs) increased the percentage of ramified microglia (Fig. 6C) up to 54.0 ± 2.6% in comparison to untreated injured animals (SCI) in these areas, thereby creating a larger anti-inflammatory microglial population (Fig. 6D). Overall, these findings suggest that NPC transplantation prompts a shift in microglial morphology in the NeuN + cell microenvironment within the injured spinal cord. Strikingly, dual treatment with NPCs and ESI-05 (SCI + NPCs + ESI-05) partially reversed the effect of NPCs on microglial status (resulting in a population of 41.9 ± 5.4% ramified-like cells), which suggests that EPAC2 mediates the NPC-induced alterations in the microglial population (Fig. 6C, D). Accordingly, animals receiving NPC transplantation (SCI + NPCs) displayed a reduction in the area positive for P2X4 receptor expression (Fig. 6E), which becomes upregulated in pathological conditions in activated microglia/macrophages [60], when compared to injured untreated animals (SCI) or animals receiving NPC transplantation and ESI-05 treatment (NPCs + ESI-05).

### EPAC2 mediates NPC transplantation-related neuroprotection after SCI

Finally, we measured the gap between rostral and caudal NeuN + cells across the injury to evaluate the effects of various treatments on neuronal sparsity caused by SCI. Interestingly, NPC transplantation (SCI + NPCs) significantly reduced the gap between NeuN + cells (Fig. 6F) and increased the density of NeuN + cells encountered in the areas surrounding the injury epicentre (Fig. 6G) compared to injured untreated animals (SCI). Of note, animals receiving NPC transplantation and ESI-05 treatment (NPCs + ESI-05) exhibited a similar gap distance and NeuN density to injured non-transplanted animals (SCI), thereby demonstrating the involvement of EPAC2 in NPC-mediated neuroprotection (Fig. 6F, G).

## Discussion

The primary injury to spinal cord tissues initiates an interconnected and evolving molecular cascade of secondary events [32], with transcriptional analysis providing a comprehensive view of the molecular mechanism underlying SCI pathophysiology [61, 62]. Advanced technologies for transcriptional studies applied to the SCI field, which include RNA-sequencing [62, 63], single-cell analysis [64], and/or axoplasmic fractioning [65], have provided novel insight into the cellular and molecular behaviour of the spinal cord under physiological or injured conditions. Here, we present a transcriptional analysis of spinal cord homogenates, which provides tissular (though not cell-type specific) information regarding critical time points after SCI (from early subacute to late chronic stages). Our approach provides wide temporal coverage to allow the study of the temporal dynamics of SCI evolution. Furthermore, we evaluated the impact of intramedullary acute or subacute transplantation of NPCs on transcriptional profiles within the SCI to define the functional outcomes of NPC therapy.

Whole spinal cord differential transcriptomic analysis revealed two critical issues—(i) the substantial number of DEGs found after SCI demonstrates the strong transcriptional impact of SCI (affecting up to 55.48% of the total transcripts), and (ii) most DEGs (up to 9993) display commonality at all time points under study (from 1 to 8 weeks after SCI) indicating a lack of efficient transcriptional resolution. Nevertheless, comparisons made during injury progression established that the most significant number of DEGs occurred between the first and the second weeks after SCI; therefore, early therapeutic interventions may prevent later transcriptional dysregulation. Transcriptional characterization indicated that NPC transplantation transcriptionally modulates those SCI-related and non-SCI-related genes necessary for injury resolution. Interestingly, NPC-related DEGs comprise functional blocks relevant to CNS regeneration, such as neural function, immune response, or ionic homeostasis.

Temporal clustering provided evidence that functions such as "G-protein coupled glutamate receptor signalling" and "Adenosine Receptor signalling pathway" (GO:0,007,216; listed in cluster 3) became upregulated during the first 4 weeks after SCI before returning to preinjury levels by week 8. The transcriptional upregulation of genes implicated in glutamate receptor transduction may be a consequence of the high availability of this neurotransmitter during SCI. Similarly, the elevated levels of extracellular ATP observed after SCI (which undergoes metabolism to form adenosine [66]) may cause the overactivation of adenosine receptors and thus lead to the upregulation of related signalling components. Interestingly, in close temporal convergence, we discovered the downregulation of the "Cellular response to cAMP" within the first 4 weeks after SCI before recovering at chronic stages. Given the temporal overlap between the upregulation of glutamate and adenosine receptor cascade with the downregulation of the cAMP response, we suggest the implication of glutamate and ATP leakage in cAMP decay after SCI through Gi protein activation linked to metabotropic glutamate receptors or adenosine receptors [67, 68]; however, we note the need for further experimental validation to fully explore and validate this hypothesis.

We also note the recurrent appearance of cAMP-associated GO terms among altered functions after SCI. Reports have described the regenerative efficacy of cAMP-directed therapies in SCI and provided evidence for the involvement of distinct mechanisms. For instance, cAMP treatment helped axons to overcome growth inhibition by the myelin-associated glycoprotein (MAG) [69, 70] and promoted axon regeneration in the spinal cord, as shown by the capacity of intraganglionic dibutyryl cAMP (db-cAMP) microinjections to induce the growth of injured sensory branches [71]. Similarly, in vivo imaging of fluorescently labelled reticulospinal axons of injured lampreys demonstrated that treatment with a cAMP analogue inhibited axon retraction and enhanced axon growth [72]. Furthermore, db-cAMP treatment induced axons to grow in straighter paths and increased the number of surviving axotomized neurons [19, 73]. Importantly, these in vivo studies apply general methods that prompt cAMP accumulation, which impedes the exploration of the specific involvement of EPAC2 or PKA on cAMP-related effects in spinal cord regeneration; however, Lin et al. reported that the cortical infusion of a selective PKA antagonist enhanced functional improvements after rehabilitative training in injured rats after SCI (contrary to their initial hypothesis) [74]. This finding suggested EPAC2 as a mediator of cAMP-derived-neuronal regeneration and neuroplasticity in the context of SCI for the first time.

cAMP signalling begins by cAMP binding to PKA or EPAC2 and the subsequent activation of distinct signalling pathways, which gives rise to the duality of EPAC2 and PKA functions. The differential activation of PKA and EPAC2 may derive from the regulatory activities of intracellular cAMP levels and the prolongation of cAMP stimulation [75]. Since both PKA and EPAC2 play relevant neuroregenerative functions, we believe that PKA/EPAC2 interplay may have significant implications for SCI; therefore, a deeper understanding of this process remains of great interest.

Although PKA and EPAC2 trigger CREB phosphorylation through MAPK signalling, they possess differential mechanisms of action; for instance, PKA activation in cultured dentate granule cells promotes axonal branching, while EPAC2 activation promotes axonal elongation [53]. EPAC2 participates in a wide range of neural functions, including axonal growth and guidance [21], synapse remodelling through Rap-dependent AMPA GluR2/3 removal [69], and attenuation of microglial and astrocytic activation [22]; however, EPAC2-specific functions remained relatively unexplored in the context of SCI. We employed a combination treatment comprising a specific EPAC2 inhibitor (ESI-05) *and* NPC transplantation to investigate any possible EPAC2-dependent neuroprotective and neuroregenerative capacities. Although ESI-05 targets EPAC2 protein activity, qPCR and immunohistological analysis indicated that the sustained inhibition of EPAC2 resulted in the downregulation at the protein and mRNA levels.

Previous reports indicated that cAMP induction modulated astrocytic reactivity and reduced scar tissue in vivo [25] and that the specific activation of EPAC2 within the lesion in an ex vivo model of SCI resulted in the generation of astrocytes with elongated processes that were permissive to axon regrowth [24]. We evaluated whether NPC transplantation and EPAC2 inhibition by ESI-05 impacted the extension of GFAP delimited scars. NPC transplantation reduced the extension of GFAP- areas 2 weeks after SCI; however, cotreatment of NPCs with ESI-05 increased the scar area to a level similar to that observed in injured non-transplanted animals. NPCs may serve as an extra supply of astrocytes [13] or induce astrocyte mobilization in the initial stage of glial scaring, which is essential for optimal injury preservation of the remaining healthy tissue (reviewed by Falnikar, Li, and Lepore [76]). Overall, our results indicate that EPAC2 inhibition during NPC transplantation may impede gliogenic potential and avoid appropriate astroglial scaring, prompting lesion enlargement. In agreement, cortical NPCs obtained from EPAC2-knockout mice cannot undergo astrocytogenesis induced by pituitary adenylate cyclase-activating peptide (PACAP) [55]. Although our results suggest a role for EPAC2 in the initial formation of the astrocytic border, we hope that further investigations during chronic scenarios will provide further insight.

We morphologically evaluated Iba-1 + cell morphology as an indicator of microglial phenotype in our in vivo model of SCI; specifically, we analysed NeuN + rich areas, considering the high relevance of the microglia-neuron interaction. Morphological analysis indicated that NPC transplantation increased the percentage of ramified microglia compared to injured non-transplanted animals, suggesting that NPC transplantation could polarize microglia into an anti-inflammatory state (thereby providing a more permissive environment) in neuron-rich areas through an EPAC2-associated mechanism. Previous observations supporting these results described how co-culturing NPCs with brain slices induced neuron survival through microglial polarization into an anti-inflammatory phenotype, which included a reduction in tumour necrosis factor-alpha (TNFα) secretion and an increase in insulin like-growth factor 1 (IGF-1), chemokine (C-X3-C motif) receptor 1 (CX3CR1), and triggering receptor expressed on myeloid cells 2 (TREM2) secretion [77]. Interestingly, ESI-05 administration reversed the effect of NPC transplantation on microglial polarization, thereby suggesting the involvement of EPAC2. Similarly, previous data noted that EPAC2 activation with S-220 in lipopolysaccharides (LPS)-stimulated microglia significantly reduced their inflammatory-like activation status by reducing inducible nitric oxide synthase (iNOS) expression and NO release in vitro, which led to a decreased cell body perimeter that resembled the non-injured morphology in an ex vivo model of SCI [24].

We also employed immunostaining to demonstrate that NPC transplantation decreased P2X4 levels, which agrees with results from a previous study [78], and that cotreatment with an EPAC2 inhibitor avoided this effect. A previous study reported that P2X4 receptor levels increase after SCI upon microglia/macrophage activation [60], while others have shown that P2X4 receptor expression promotes local inflammatory responses and neuropathic pain [60, 79]. Thus, the decrease in P2X4 receptor expression mediated by NPC transplantation suggests that NPC therapy results in an anti-inflammatory outcome. The reversion of P2X4 receptor expression status after ESI-05 treatment suggests a dependency on EPAC2 for the anti-inflammatory activities exerted by administered NPCs.

In addition to the attenuation of the proinflammatory environment, we also aimed to explore whether NPC transplantation altered neuropreservation in injured tissue by measuring the distance between NeuN + cells across lesions. Interestingly, we discovered that NPC transplantation favoured neuropreservation, with a two-fold lower gap distance between NeuN + cells and higher NeuN + density proximal to the injury epicentre observed. A reduction in the distance between neurons across the injury may indicate a more neuropermissive microenvironment and/or represent a crucial stage in facilitating neuronal plasticity and the formation of new relays, as rostral regenerating axons will cross shorter distances to reach caudal targets. Of particular interest, ESI-05 treatment reversed NPC-mediated neuroprotection, supporting a similar NeuN + cell gap observed in injured non-transplanted animals. A review of the literature provided evidence of some debate regarding the role of EPAC2 in mediating neuronal apoptosis/survival. While some studies indicate the involvement of EPAC2 in p38-mediated neuronal apoptosis in traumatic brain injury [80], others report improved locomotor recovery after S-220 treatment for SCI, thereby suggesting a neuroprotective role of EPAC2. The regulation of apoptosis or survival via EPAC2 may depend on different scenarios of neural injury pathogenesis [81]. In the context of SCI, we believe that EPAC2 mediates neuronal survival by globally impacting the tissue environment to reduce scar expansion and local proinflammatory processes mediated by M1-polarized microglia. Thus, the detrimental contribution of ESI-05 on the neuroprotective effect of NPCs may derive from an aggressive micro environmental context due to lesion expansion and exacerbated microglial proinflammatory responses caused by an absence of EPAC2.

Considering the short-time window of NPC intervention (1 week after transplantation), the ability of NPCs to provide trophic factors into the injured environment (as previously described [15]) should have relevant contribution in their therapeutic effects. Grafted NPCs constitutively secrete neuronal growth factor (NGF), brain and glial-derived neurotrophic factors (BDNF, GDNF) in vivo to inhibit neuronal loss after secondary excitotoxic damage, reduce the activation of both microglia and astrocytes, and impact the growth of sensory and motor axons, which are sensitive to those cues [15].

Overall, our data suggest that NPC transplantation promotes a more neuropermissive microenvironment during SCI by reducing scar extension, attenuating microglial activation, and promoting neuropreservation through EPAC2-dependent mechanisms.

### Conclusions

Overall, we provide a comprehensive, wide-ranging temporal profile of the physiopathological evolution of SCI combined with a transcriptional characterization of the impact of NPC transplantation. We have deposited the transcriptional data sets generated in the GEO under accession code GSE183591. We also propose a new hypothesis for cAMP decay after SCI, which may provide the impetus for additional research. We describe the tissue-specific and temporal expression of cAMP signalling components after SCI and experimentally demonstrate that NPC transplantation counteracts a significant proportion of SCI-induced cAMP signalling dysregulation. Finally, we demonstrated the influence of EPAC2 on the immunomodulatory and neuroprotective outcomes associated with NPC transplantation.

## Methods and materials

### Spinal cord injury model

Sprague Dawley rats (~ 200 g) were bred at the Animal Experimentation Unit of the Research Institute Príncipe Felipe (Valencia, Spain). Only female rats were used due to the ease of manual bladder emptying required after SCI, the reduced frequency of urinary infections after SCI [82], and more stable body weight during the experimental timeframe [83] compared to male rats. The maintenance and use of all animals were in accordance with guidelines established by the European Communities Council Directive (86/609/ECC) and the Spanish Royal Decree 53/2013. All experimental procedures were approved by the Animal Care and Use Committee of the Research Institute Prince Felipe (2021/VSC/PEA/0032). Rats were housed under standard temperature conditions with controlled 12 h light/dark cycles with ad libitum access to food and water. All animals were maintained by professionally trained staff.

For the surgical interventions, female rats were subcutaneously pre-medicated with morphine (2.5 mg/kg) and anesthetized with 2% isoflurane in a continuous oxygen flow of 1 L/min. Laminectomy was performed of the thoracic vertebrae 8–9 to expose the spinal cord and induce severe SCI at thoracic vertebrae 8 level by contusion applying 250 kdyn force using Infinite Horizon Impactor, as previously described [7, 84]. All animals were subjected to post-surgery care consisting of manual drainage of bladders twice a day until vesical reflex was recovered and subcutaneous administration of 5 mg/kg of Enrofloxacin (Alsir) for 7 days, 0.1 mg /kg of Buprenorphine twice a day for 4 days after each intervention, and 1 mg/kg of Tacrolimus starting from one day before NPC transplantation until the experimental endpoint. The experimental protocol included humane endpoint criteria when severe signs of distress were observed.

### NPC culture and transplantation

NPC isolation and culture were performed as previously described [85]. Briefly, spinal cords were harvested from adult (for the microarray performance) or neonatal (for the qPCR validation and combinatory transplantation with ESI-05 or vehicle) spinal cords (P3P5) in cold Hank's balanced saline solution (HBSS) with 10 µl/L penicillin/streptomycin (P/S; Sigma). Dissected tissue was gently pipetted to induce mechanical disaggregation, and cells were then cultured on ultra-low attachment plates as neurosphere-like cultures. Proliferation culture media comprised NeuroCult™ Proliferation Medium (StemCell Technologies, Grenoble, France) supplemented with NeuroCult™ Proliferation Supplement (Stem Cell Technologies) and 0.7 U/mL heparin (Sigma), 10 µl/L P/S (Sigma), 20 ng/mL epidermal growth factor (EGF; Thermo Fisher, Horsham, UK), and 20 ng/mL basic fibroblast growth factor (bFGF; Invitrogen). NPC transplantation was carried out directly after (acute model) or 1 week after (subacute model) contusion. 1 × 10^6^ NPCs at passages 3–4 were intramedullary administered at two injection points (2 mm caudal and rostral to the lesion epicenter) using a 26 G Hamilton pipette coupled to a siliconized glass pipette containing the cell suspension. NPCs were infused at a rate of 2 µl/min in a total volume of 5 µl/injection, waiting 2 min before removing the syringe to allow cell deposition into the tissue (as previously described [84]). Control injured animals were injected with the same volume of the cell-free medium. All animals were sacrificed 1 week after transplantation.

For the transcriptional study, animals were distributed into the following groups: animals sacrificed at one (T1), two (T2), four (T4), or eight (T8) weeks after SCI; and SCI animals with NPC transplantation at acute (T1_NPCS_) or sub-acute stages (T2_NPCS_) (*n* = 8 animals/group; *n* = 4 for microarray performance and *n* = 4 for qPCR validations). The in vivo combinatory treatment of NPC and the EPAC2 inhibitor (ESI-05) employed the following groups: SCI, SCI + NPCs, and SCI + NPCs + ESI-05 (*n* = 3 animals/group).

### Drug administration

Drug administration was performed by intrathecal catheterization starting 1 week after injury. Partial laminectomy and dura mater perforation were performed at T13 to introduce the catheter (Alzet Corp., Cupertino, CA, USA; previously filled with 0.9% saline solution) to the injured segment (Thoracic vertebrae 8 level). Catheters were coupled to osmotic pumps (Model 1007D, Alzet Corp. Germany) previously filled with 100 μl of drug solutions (either DMSO 60% [vehicle] for SCI and SCI + NPCs animals or ESI-05 12 mM in DMSO 100% for SCI + NPCs + ESI-05 animals) and incubated overnight at 37 ºC in a saline solution following the manufacturer's instructions. Pumps delivered 0.5 µl/h for 1 week to achieve a sustained concentration of 15 µM of ESI-05 (assuming a cerebrospinal fluid volume of 400 µl after injury).

### Sacrifice and sample processing

Animals were overdosed with sodium pentobarbital (100 mg/kg) and transcardially perfused with 0.9% saline solution. For further processing, spinal cord tissue was carefully removed and dissected into rostral, injured, and caudal sections (as described in each experimental design, see Figs. 1A, 4A, and 5A). Samples destined for histological analysis were fixed by immersion in 4% paraformaldehyde in phosphate buffer saline (PBS) for 24 h and then maintained in 0.1 M phosphate buffer (PB). Samples destined for RNA and protein isolation, obtained from 0.5 cm of the tissue adjacent (caudal and rostral) to the injury epicentre, were frozen in liquid nitrogen and stored at – 80 ºC until use.

### Microarray analysis

Total RNA was extracted using the RNeasy Mini Kit (QIAGEN) for transcriptional analysis according to the manufacturer's instructions. Labelled cRNA (3 µg) was hybridized with the Whole Rat Genome Oligo Microarray Kit (Agilent p/n G2519F-014879). The protocol and the raw and normalized data were deposited in the Gene Expression Omnibus (GEO) as open access at www.ncbi.nlm.nih.gov/geo/query/acc.cgi?acc=GSE183591.

### Data processing and bioinformatic workflow

Agilent raw data were pre-processed by normalization and background correction using Agilent methodology. The intensity signal was standardized across arrays via quantile normalization [86].

The normalized expression matrix was used as input for two different analyses.

1. Differential expression analysis: Pair-wise analyses between conditions were designed to study: (i) the temporal evolution of the injury (every time point condition (T1–T8) versus uninjured (T0): T1–T0; T2–T0; T4–T0; T8–T0, (ii) the progression evaluation (each time point condition versus its previous time point), and (iii) treatment intervention (acute and subacute transplantation versus untreated and early subacute or late subacute SCI animals). Then, differential gene expression assessment for all comparisons was carried out using limma moderated *t*-statistics [87]. Multiple testing p-value corrections used the Benjamini–Hochberg procedure to derive adjusted *p*-values. Genes were called differentially expressed if their corrected *p*-value was smaller than 0.05.
2. Functional analysis: pathway analysis using the Hipathia R/Bioconductor package was first performed to estimate the level of activation of each subpathway in every sample from gene expression data. The effector proteins of a subpathway can be linked to GO terms (Biological Processes); Hipathia computes the level of activation of each function, summarizing the activity of all subpathways annotated in a GO term. Finally, a function activity analysis for all comparisons was performed using the limma package. The *p*-values were adjusted using the Benjamini–Hochberg method [34]. Those functions with an adjusted *p*-value lower than 0.05 were considered significant.

All bioinformatic steps were performed with the programming language R. Principal Component analysis was performed using the PCA function from the mixOmics package. Unsupervised hierarchical clustering heatmaps (using the euclidean distance) were generated with the heatmaply package.

### RNA extraction and RT-qPCR

Tissue samples were mechanically disrupted in 1 ml of TriZol Reagent (Invitrogen, Massachusetts, USA) using an ultraturrax. RNA extraction was performed according to Trizol standard method and afterward submitted to an additional step of clean-up by RNeasy MinElute Cleanup (Qiagen, Germany) to ensure the quality of the sample (*A*_260/280_ ≈ 2 and *A*_260/230_ ≥ 1.8).

One  μg of total RNA was reverse-transcribed using the high-capacity RNA-to-cDNA™ kit (Applied Biosystems, Massachusetts, USA) in a 30 min reaction at 42ºC. Specific primers were designed for each gene of interest- (Online Resource Table 5) using primer-BLAST (NCBI, Maryland, USA) and evaluated by efficiency curve performance. qPCR was performed in triplicate using AceQ SYBR qPCR Master Mix (ThermoFisher) in the Light-Cycler 480 detection System (Roche, Basel, Switzerland). Ct data were obtained with the LightCycler 480 relative quantification software (Roche, Basel, Switzerland). GAPDH mRNA level was used as an internal control for normalization. Changes in expression relative to control samples were calculated as 2^−ΔΔCt^.

### cAMP determination

 ~ 20 mg of each frozen tissue sample from rostral and caudal regions was weighed and homogenized in 10X volume of 0.1 M HCl. Determination of cAMP was performed using cAMP Direct Immunoassay Kit (ab65355, Abcam) following the manufacturer's instructions. The absorbance of each sample (in duplicate) at OD_450nm_ was measured by a VICTOR2 D fluorometer (PerkinElmer). The calculation of cAMP concentration was assessed by extrapolation to a standard curve.

For the cAMP analysis in the injury site, three longitudinal slices per animal in mid-depth of the spinal cord (taking the central canal as reference) were immunoassayed. The area positive for cAMP signal was normalized to the slice's total area.

### Histological assessment

After carefully removing the meninges, tissues were placed into paraffin blocks using a Modular tissue embedding centre (Myr, SL). Spinal segments were cut into longitudinal sections of 7 μm thickness using an HM340E Electronic rotary microtome (VWR International, Barcelona, Spain).

Paraffin tissue slices were first baked and washed. Prior to immunofluorescence, slices were incubated at 97ºC for 25 min in Tris–EDTA Buffer (10 mM Tris Base, 1 mM EDTA Solution, 0.05% Tween 20, pH 9) to unmask the epitopes and then blocked and permeabilized with 0.1% Triton X-100, 5% horse serum, and 10% foetal bovine serum in PBS. Immunofluorescence double staining was performed by overnight incubation at 4 ◦C with primary antibodies and 1 h with secondary antibodies at room temperature.

Primary antibodies used were—anti-B-III-Tubulin (α-ms,1:400, MO15013, Neuromics, Edina, MN, USA), anti-NeuN (α-CK,1:600, ABN91, Merck Millipore, Massachusetts, USA), anti-GFAP (α-CK, 1:1000 PA1-10,004, Thermo Fisher, Massachusetts, USA), anti-cAMP (α-Rb,1:100, 07–1497, Merck Millipore, Massachusetts, USA), anti-Epac2 (α-Rb,1:400, 43,239, Cell Signalling Technology, Massachusetts, USA), Iba-1 (α-Rb,1:400, 019–19,741, WAKO, Osaka, Japan), P2X4 (α-Rb, 1:400, purchased from Alomone Labs, Jerusalem, Israel). Secondary antibodies used were either AlexaFluor-488, -555, or -647 (1:400, Invitrogen, Massachusetts, USA) conjugated antibodies against the respective IgG of the primary antibody. DAPI (1:1000, Sigma, Missouri, USA) was used to stain cell nuclei.

Fluorescence images of each spinal cord slice were acquired using an Aperio Versa scanner (Leica Biosystems, Wetzlar, Germany). Images were visualized and quantified with the Image Scope software or ImageJ/Fiji software.

### Analysis of GFAP- areas

The glial scar is delimited by border-forming astrocytes expressing GFAP [88]. The analysis of the extension of the scar was carried out by quantifying the area delimited by GFAP staining (GFAP negative area) in at least twelve longitudinal slices per animal in a central range of – 252 to + 210 microns from dorsal to ventral, including the central canal (0), as represented in the illustration in Fig. 6A.

### Analysis of microglial circularity and P2X4 expression

Iba-1 immunostaining was employed to study the microglial morphology associated with their inflammatory profile [59]. As Iba-1 can also label engrafted macrophages, microglial populations within NeuN + dense areas of the white matter (which differ from the tissular location where the macrophages are found [into and surrounding injury zone]) were selectively studied.

ImageJ was used to compute the circularity of each Iba + cell in two different slices obtaining populations of more than 3800 cells per animal. Microglial cells were categorized by their activation level into subpopulations according to their circularity: (i) ≤ 0.25 (circular microglia) and (ii) > 0.25 (ramified microglia). P2X4 quantification employed the analysis of spinal cord slices, including the injury epicenter, by calculating the percentage of P2X4 + staining/total spinal area for each animal, as represented in Fig. 6E.

### Analysis of NeuN + Cell gap

The distance between NeuN + cells across the injury site was measured to evaluate the sparsity of neurons caused by SCI. Additionally, the number of NeuN + cells in tissue ranges of 2 mm from the injury epicenter towards rostral or caudal was counted, and the total number of NeuN + cells detected in each range per animal was calculated. The heat map representation in Fig. 6G shows the mean value per group. Twelve spinal cord slices per animal in a central range of − 252 to + 210 microns from dorsal to ventral, including the central canal (0).

### Statistical analyses

Bar plots are represented as mean ± standard error mean (SEM), and data were statistically analysed by Graph Pad Prism Software. First, the normality test Shapiro–Wilk was performed to test for the Gaussian distribution of each data set. If normality was met, homoscedasticity was evaluated using Bartlett's test. Heat maps, clustering graphics, and dot plots were generated with R. Comparisons between two groups used a one-tailed t-test with a confidence level of 95%. Comparisons between more than two groups were performed using a one-way ANOVA and Bonferroni's post-test for normal and homoscedastic data sets. If normality was not met, Kruskal–Wallis One-way ANOVA with Dunn's method or Tukey test post hoc correction was used. In the case of repeated measurements per variable, a nested ANOVA was performed. In the case of repeated measurement throughout the spinal depth, we used a two-way ANOVA and Tukey post-test.

## Supplementary Information

Below is the link to the electronic supplementary material.

Supplementary file1 (XLSX 847 KB)

Supplementary file2 (TXT 4 KB)

Supplementary file3 (XLSX 39 KB)

Supplementary file4 (DOCX 17 KB)

Online Resource Figure 1: Cumulative number of DEGs during SCI progression. The cumulative identification of DEGs at each time point after SCI represents the total number of genes dysregulated by SCI (even given correction at later points)

Online Resource Figure 2. Temporal clusters of altered biological functions after SCI. Graphical representation of the temporal pattern after SCI for each cluster and graphical representation of the activation level of highlighted functions for each cluster (except clusters 3 and 7, see Figure 2B)

Online Resource Figure 3. Venn diagram depicting DEGs impacted by acute (blue) and subacute (green) NPC transplantation and their intersection with SCI-related DEGs at one (yellow) and two (red) weeks after injury

Online Resource Figure 4. Highlighted GO terms altered by NPCs. GO terms altered by acute or subacute NPC transplantation and arranged by functional blocks have been selected

Online Resource Figure 5. EPAC2 staining in somas. (A) Representative images showing double immunostaining of EPAC2 and B-III-Tubulin cells. Two distinct types of EPAC2 labeling are detected: cytoplasmic (*) or plasma membrane-associated (◄). (B) Quantification of the percentage of cells with EPAC2 staining in the plasma membrane (p.m-EPAC2+ cells) showed no significant differences among groups

## Data Availability

The data that support the findings of this study are available on request from the corresponding author. The protocol and the raw and normalized data were deposited in the GEO for open access at https://www.ncbi.nlm.nih.gov/geo/query/acc.cgi?acc=GSE183591, with the accession number GSE183591.
