# Supplementary material for: NPC transplantation rescues sci-driven cAMP/EPAC2 alterations, leading to neuroprotection and microglial modulation
Source: Cell Mol Life Sci. 2022 Jul 29;79(8):455. doi: 10.1007/s00018-022-04494-w (PMC9338125; doi:10.1007/s00018-022-04494-w)
Supplement: Supplementary file 4 — Supplementary file4 (DOCX 17 KB) [file 18_2022_4494_MOESM4_ESM.docx]

| **GEN** | **PRIMER** | **PRIMER (5’-3’)** |
| --- | --- | --- |
| **EPAC2** | FW | ACAATACGCCTTGAGCCATCT |
|  | RV | GAGAAGGCTGTGCGTGGTAA |
| **BDNF** | FW | AGCAGTCAAGTGCCTTTGGA |
|  | RV | GACATGTTTGCGGCATCCAG |
| **PKA** | FW | TGCCACAACTGACTGGATCG |
|  | RV | GGCACAAGCACACCCCTAAA |
| **CaMKK2** | FW | AGAACTGCACACTGGTCGAG |
|  | RV | CCGGCTACCTTCAAATGGGT |
| **DARPP32** | FW | AGCAACCTGAGTGAGAACCAG |
|  | RV | AAGTAAGCTTCTGCCCCGC |
| **CREB1** | FW | CAGACAACCAGCAGAGTGGA |
|  | RV | TACAGTGGGAGCAGATGACG |

**Supplementary Material 1**. Primers sequences used for RT-qPCR analysis. FW: Forward primer; RV: Reverse primer; EPAC2: Exchange protein activated by cyclic AMP 2; BDNF: Brain Derived Neurotrophic Factor; PKA: Protein Kinase A; CAMKK2: Calcium/Calmodulin Dependent Protein Kinase Kinase 2; DARPP32: dopamine and cAMP-regulated phosphoprotein Mr 32; CREB: cAMP Responsive Element Binding Protein 1
