# Supplementary figures and images for: NPC transplantation rescues sci-driven cAMP/EPAC2 alterations, leading to neuroprotection and microglial modulation
Source: Cell Mol Life Sci. 2022 Jul 29;79(8):455. doi: 10.1007/s00018-022-04494-w (PMC9338125; doi:10.1007/s00018-022-04494-w)

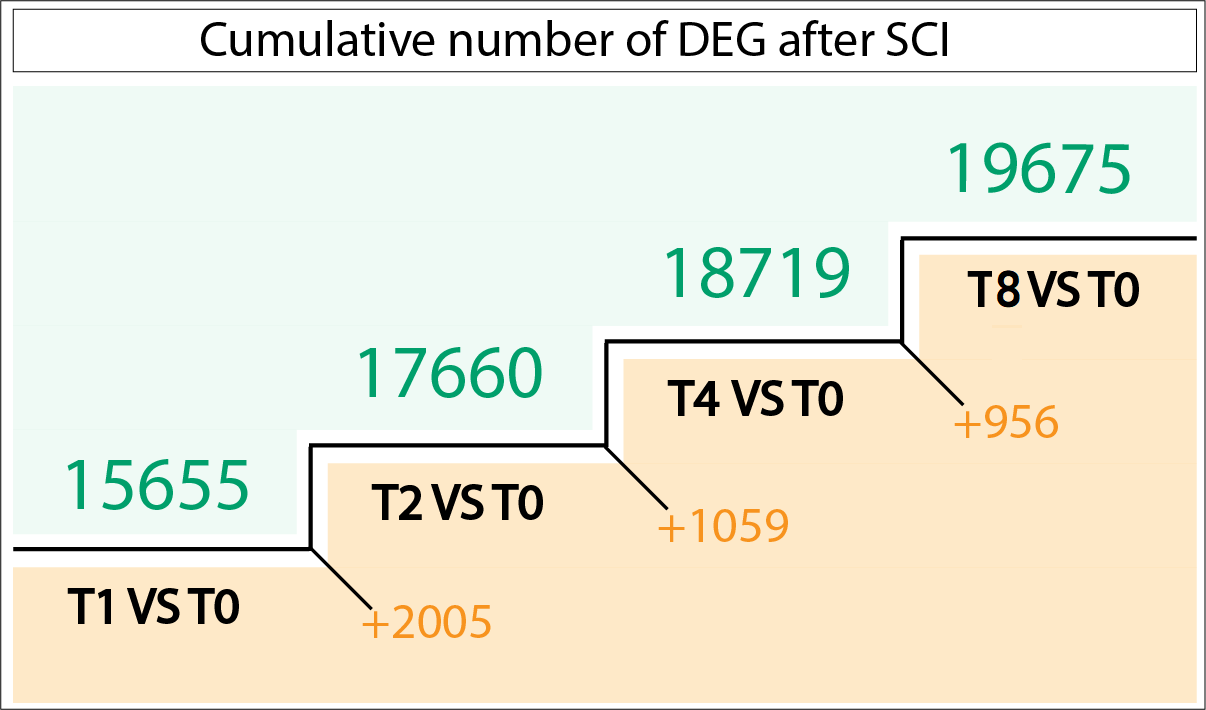

Supplement: Supplementary file 5 — Online Resource Figure 1: Cumulative number of DEGs during SCI progression. The cumulative identification of DEGs at each time point after SCI represents the total number of genes dysregulated by SCI (even given correction at later points) [file 18_2022_4494_MOESM5_ESM.png]

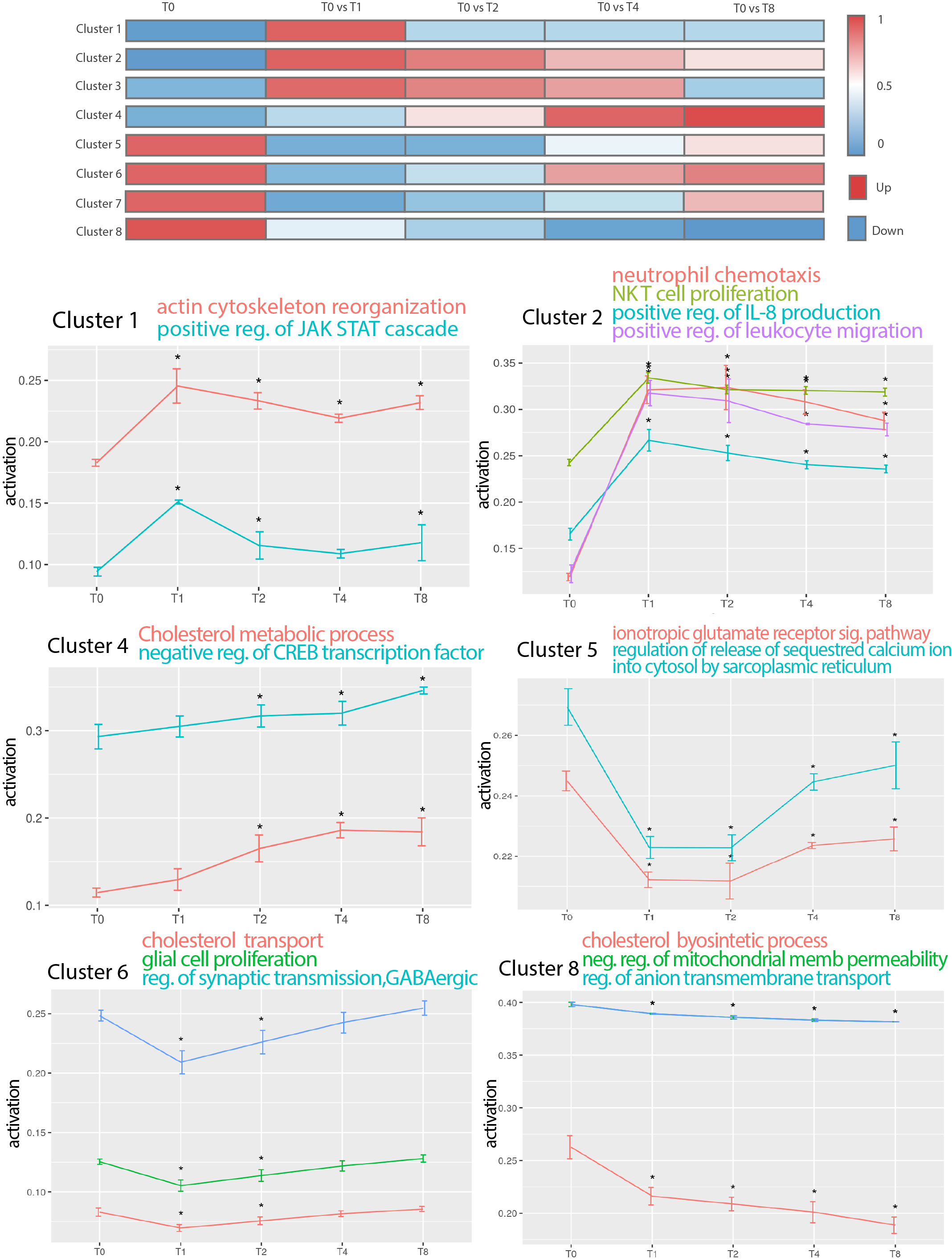

Supplement: Supplementary file 6 — Online Resource Figure 2. Temporal clusters of altered biological functions after SCI. Graphical representation of the temporal pattern after SCI for each cluster and graphical representation of the activation level of highlighted functions for each cluster (except clusters 3 and 7, see Figure 2B) [file 18_2022_4494_MOESM6_ESM.png]

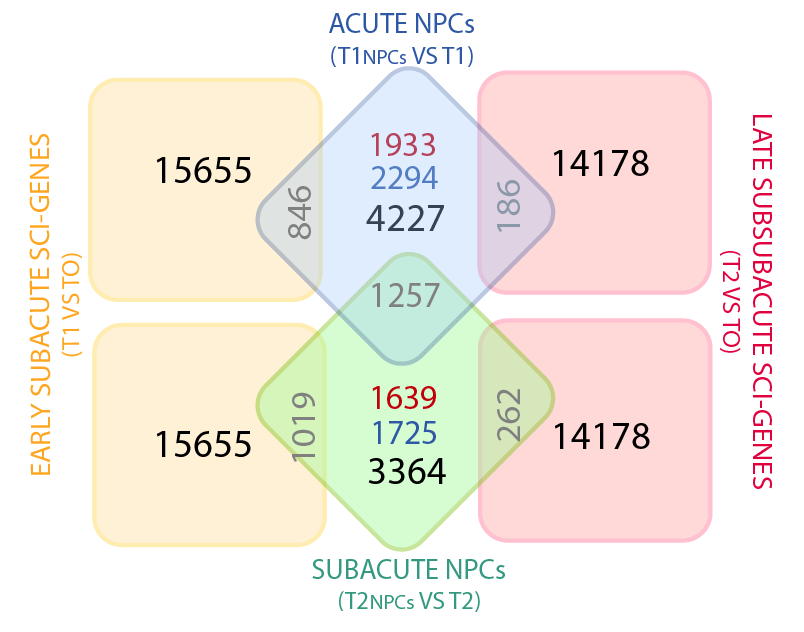

Supplement: Supplementary file 7 — Online Resource Figure 3. Venn diagram depicting DEGs impacted by acute (blue) and subacute (green) NPC transplantation and their intersection with SCI-related DEGs at one (yellow) and two (red) weeks after injury [file 18_2022_4494_MOESM7_ESM.png]

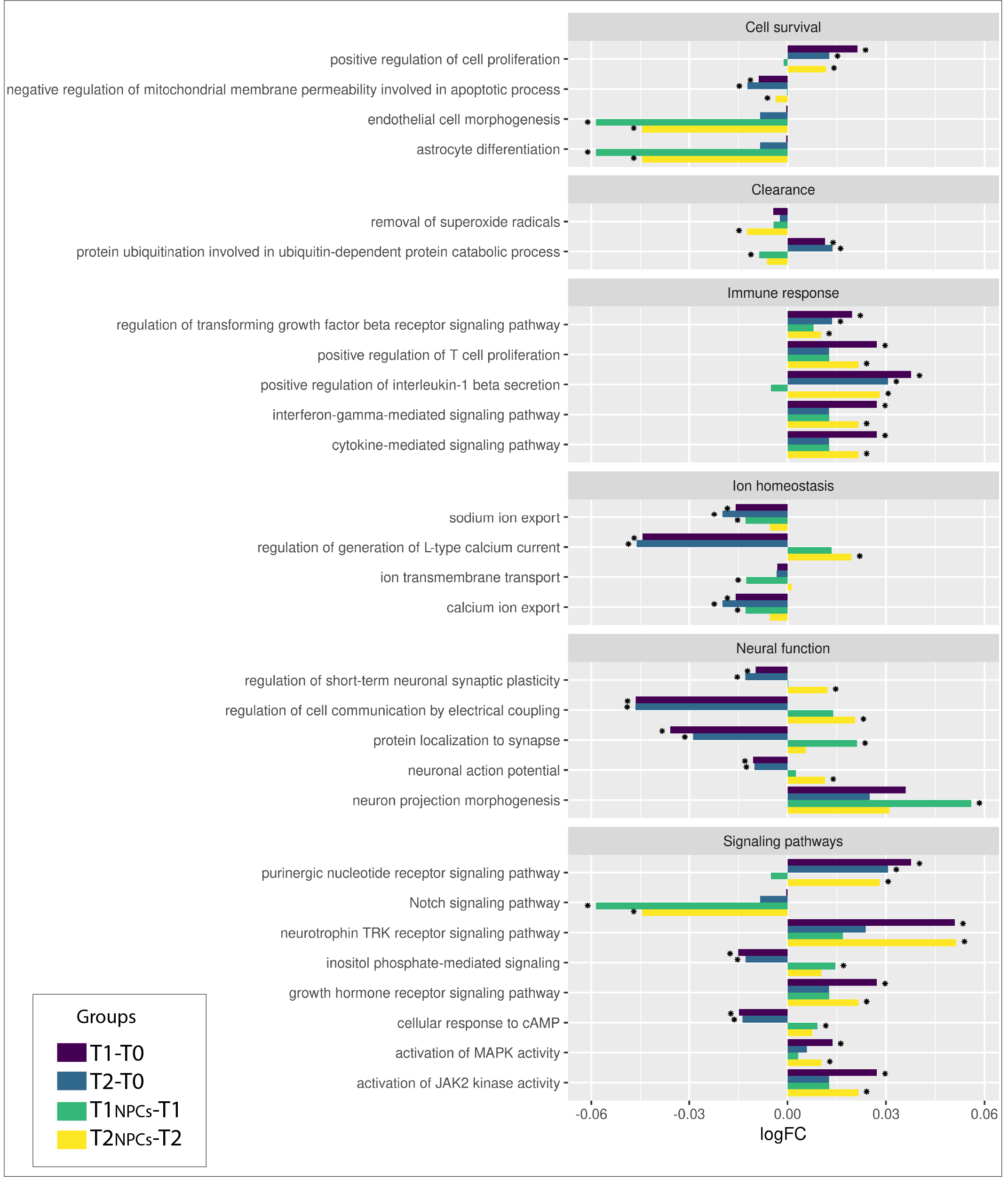

Supplement: Supplementary file 8 — Online Resource Figure 4. Highlighted GO terms altered by NPCs. GO terms altered by acute or subacute NPC transplantation and arranged by functional blocks have been selected [file 18_2022_4494_MOESM8_ESM.png]

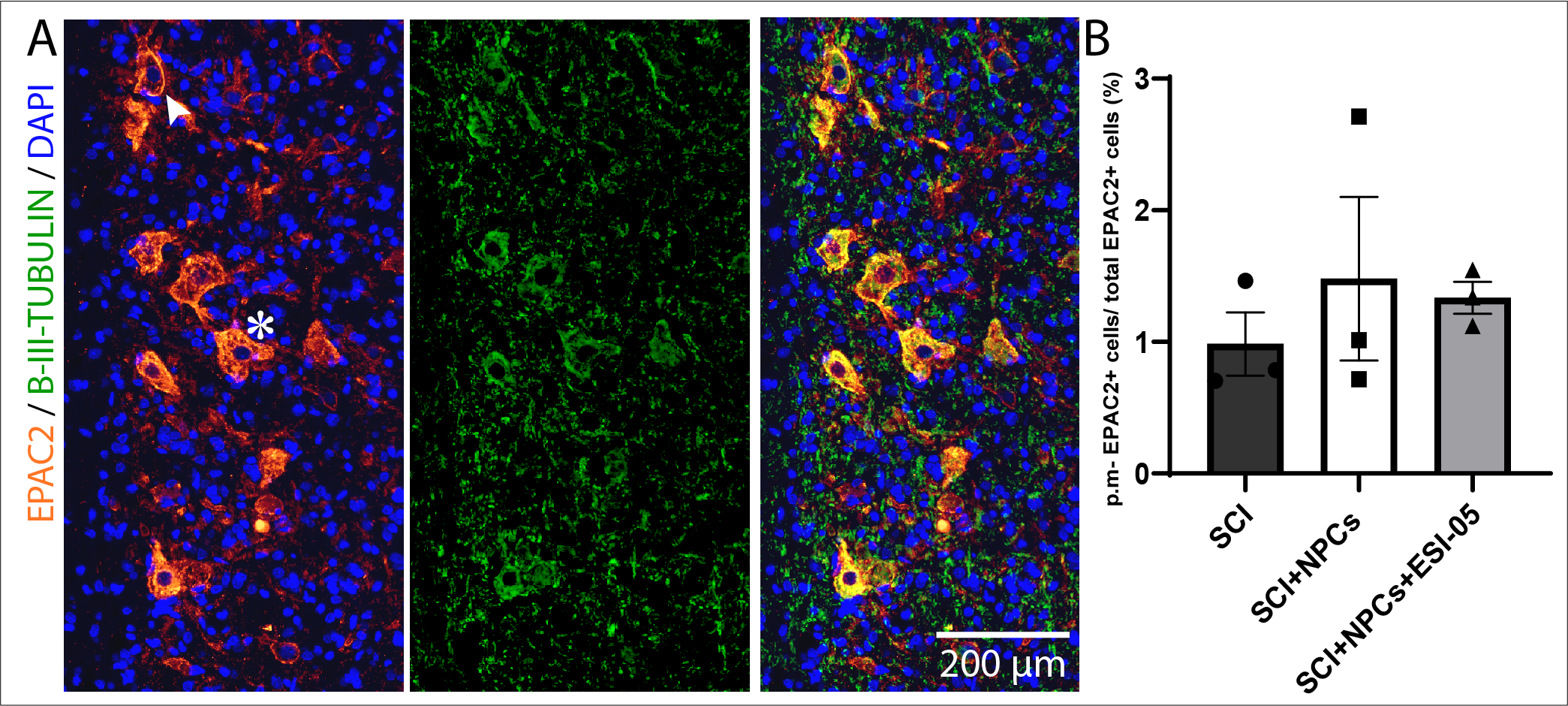

Supplement: Supplementary file 9 — Online Resource Figure 5. EPAC2 staining in somas. (A) Representative images showing double immunostaining of EPAC2 and B-III-Tubulin cells. Two distinct types of EPAC2 labeling are detected: cytoplasmic (*) or plasma membrane-associated (◄). (B) Quantification of the percentage of cells with EPAC2 staining in the plasma membrane (p.m-EPAC2+ cells) showed no significant differences among groups [file 18_2022_4494_MOESM9_ESM.png]
